# Smart greenhouse construction and irrigation control system for optimal Brassica Juncea development

**DOI:** 10.1371/journal.pone.0292971

**Published:** 2023-10-26

**Authors:** Hiep Xuan Huynh, Linh Nhut Tran, Nghia Duong-Trung

**Affiliations:** 1 College of Information & Communication Technology, Can Tho University, Ninh Kieu District, Can Tho city, Vietnam; 2 FPT Information System, Ho Chi Minh City, Vietnam; 3 German Research Center for Artificial Intelligence (DFKI), Berlin, Germany; National Technical University of Athens: Ethniko Metsobio Polytechneio, GREECE

## Abstract

This paper contributes to smart greenhouses and IoT (Internet of Things) research. Our pioneering achievement centers on successfully designing, constructing, and testing a 30m^2^ smart greenhouse, explicitly focusing on the cultivation and development of Brassica Juncea, a mustard variety commonly grown in Vietnam. The construction phase entailed the meticulous integration of diverse IoT technologies and systems, culminating in the creation of a finely tuned environment to meet the unique needs of Brassica Juncea cultivation. Notably, our research team has realized the physical infrastructure and developed and implemented a robust web interface. This interface empowers users to monitor and remotely control the smart greenhouse conveniently. It provides real-time visualization of critical parameters, including temperature, humidity, soil moisture, and light intensity, enabling precise monitoring and supporting informed decision-making in crop management. In addition to the web interface, we have meticulously designed and completed an Android mobile application, further enhancing accessibility and convenience. This mobile app allows users to monitor and control the smart greenhouse while on the move. It is imperative to underscore that this work marks a significant milestone as the first complete smart greenhouse IoT solution dedicated to developing Brassica Juncea. Our pioneering accomplishments not only advance the frontiers of innovative greenhouse and IoT research but also contribute substantially to the progress of intelligent agriculture.

## 1 Introduction

The background and significance of smart greenhouse and IoT research lie in the growing need for sustainable and efficient farming practices to address the increasing global demand for food. Traditional agricultural methods face resource utilization and environmental impact challenges, necessitating innovative solutions [1, 2]. Intelligent greenhouse systems have emerged as promising, offering benefits such as optimized resource management, improved crop productivity, and environmental sustainability. Integrating IoT technologies is crucial in enabling real-time monitoring, control, and data analysis for precise and automated management of greenhouse environments. By leveraging sensors, actuators, and communication systems, intelligent greenhouses can dynamically adjust environmental parameters based on real-time data, ensuring optimal conditions for plant growth and efficient resource utilization [3]. This research aims to contribute to the advancement of sustainable agriculture by exploring the potential of smart greenhouse and IoT integration, fostering precision farming practices, and enhancing the overall productivity and sustainability of agricultural systems [4–6].

The trend of developing intelligent products with internet connection is becoming a popular trend in the world. These products are forecasted to quickly change all countries’ economic, cultural, and social aspects. With such a boom, IoT can be used as a tool to serve agricultural and industrial production [7, 8]. The agricultural sector, which is known for its reliance on the experience of farmers, is a big challenge in finding better ways to increase the efficiency of crop production. In the era of Industry 4.0, the way that is considered the optimal and also the most irreplaceable trend is to apply new technology to production and farming activities. IoT will transform agriculture from qualitative production into precision production based on data collection, synthesis, and statistical analysis [9, 10]. It helps farmers collect crop data, such as weather conditions, soil quality, crop growth progress, or the health of cattle, and traceability through smartphones and computers. At the same time, it increases business efficiency through process automation, for example, irrigation, fertilization, pest control, reasonable water control, fertilizers, and pesticides. Thereby improving product quality, minimizing human effort, and optimizing labor productivity [11]. Some applications of IoT in agriculture are as follows.

**Precision farming** Precision farming can be considered more precise controlled farming when raising livestock and crops. In this approach to farm management, a key component is the use of information technology and various items such as sensors, control systems, robots, autonomous vehicles, automated hardware, and technology. Agriculture also uses drones for crop health assessment, irrigation, crop monitoring, pesticide spraying, crop cultivation, and soil analysis. Precision agriculture is one of the most well-known applications of IoT in agriculture, and many organizations are using this technique worldwide [12, 13].

**Livestock monitoring** Large ranchers can use wireless IoT applications to collect data on the location and health of their livestock. This information helps them identify sick animals so they can be separated from the herd, thus preventing the spread of disease. It reduces labor costs as ranchers can locate their cattle with the help of IoT-based sensors [14, 15].

**Smart greenhouse** Growing plants in a greenhouse is a method to help improve the yield of vegetables, fruits, and crops. Greenhouses control environmental parameters through manual intervention or control mechanisms. A competent greenhouse can be designed with the help of IoT, which intelligently monitors and manages the climate, eliminating the need for manual intervention. It is necessary to use different sensors that measure the environmental parameters required by the plant to control the environment in an intelligent greenhouse. We can create a cloud server for remote access to the system when connecting to IoT [16–19].

The convergence of IoT technologies with agricultural practices has led to the emergence of smart greenhouses. These intelligent structures integrate advanced sensors, automation systems, and data analytics to optimize cultivation processes and promote sustainable agriculture. By harnessing the power of IoT, smart greenhouses offer a promising solution to address the challenges of resource efficiency, climate variability, and food security in modern farming. IoT-enabled smart greenhouses go beyond traditional agricultural practices by utilizing sensor networks to collect real-time data on various environmental parameters [20]. These sensors continuously monitor factors such as temperature, humidity, light intensity, soil moisture, and carbon dioxide levels within the greenhouse. The collected data is then transmitted to a central system for analysis and decision-making [21].

Automation systems play a crucial role in intelligent greenhouses, enabling precise control and management of environmental conditions. By leveraging actuators and control algorithms, these systems regulate factors like ventilation, shading, irrigation, and fertilization. The automation reduces human intervention and ensures optimal conditions for plant growth, resulting in improved productivity and resource conservation. The IoT aspect of smart greenhouses becomes evident in the seamless connectivity between sensors, actuators, and the central control system. The greenhouse components exchange data through wireless communication protocols and receive real-time instructions. This connectivity enables remote monitoring and control of the greenhouse environment, allowing farmers to access critical information and make informed decisions from anywhere at any time [22]. The wealth of sensor data available presents a compelling opportunity to explore and develop advanced forecasting applications [23–25] in the realm of agriculture [26].

Integrating data analytics and artificial intelligence further enhances the capabilities of intelligent greenhouses. These systems can derive valuable insights and predictive models by applying advanced algorithms to the collected data. Data analytics helps farmers optimize resource allocation, improve crop management practices, and detect anomalies or diseases at an early stage. Consequently, farmers can make data-driven decisions to optimize yields, reduce waste, and enhance overall crop quality. The benefits of IoT-enabled smart greenhouses extend beyond operational efficiency. By providing a controlled environment, these structures mitigate the risks associated with external factors such as adverse weather conditions, pests, and diseases. This level of control enables year-round cultivation and reduces dependence on seasonality, enhancing food security and promoting sustainable food production practices. Smart greenhouses empowered by IoT technologies represent a significant advancement in agricultural practices. Integrating sensors, automation systems, data analytics, and connectivity offers immense potential for optimizing resource utilization, enhancing productivity, and promoting sustainable farming. By leveraging the power of IoT, smart greenhouses pave the way for a more resilient and efficient agricultural ecosystem, addressing the challenges of a growing population and a changing climate [27, 28].

The agricultural sector in Vietnam has experienced a lack of focus on research and the application of high technology in production, resulting in a lag behind global farming practices. Despite advancements in science and technology, agricultural production has yet to keep pace with these developments [29]. In light of this, our group recognized the opportunity to apply our knowledge and expertise to agricultural production by creating an automatic irrigation system incorporating the automated application of liquid fertilizer in a water tank. This system aims to reduce labor-intensive tasks for farmers, improve crop productivity, and ensure that agricultural products meet global standards. In addition to automation, the system provides recommendations on fertilizer quantities and supports the recording of fertilizer usage, enabling farmers to adhere to Good Agricultural Practices (GAP) standards. Certification under the GlobalGAP standard is internationally recognized and assures consumers and retailers that food products meet safety, quality, and sustainability criteria. It also demonstrates a commitment to worker welfare, environmental responsibility, and livestock welfare. Without meeting these standards, Vietnamese agricultural producers face difficulties accessing demanding markets such as the EU, the US, and Japan. Developing our smart greenhouse system aims to address these challenges and enhance the competitiveness of Vietnamese agricultural products in the global market.

## 2 Related work

In recent years, the agricultural sector has recognized the need for technology due to the challenges posed by climate change and resource scarcity. Farmers have been compelled to embrace innovative communication and information technologies to improve production efficiency and enhance crop resilience. Among the emerging approaches, systems engineering, and information infrastructure based on the Internet of Things (IoT) have gained significant attention. In agriculture, IoT solutions aligned with the demands of Industry 4.0 have found applicability in greenhouses [30]. Greenhouses provide controlled environments that facilitate optimal plant growth. IoT technologies for smart greenhouses encompass sensor systems, devices, and communication infrastructure, enabling real-time monitoring, data collection, and processing. This integration allows for efficient control of critical indoor parameters, including light exposure, ventilation, humidity, temperature, and carbon dioxide levels [31, 32]. In-depth discussions on IoT-based applications, sensor devices, and communication protocols for greenhouse systems can be found in the comprehensive study by Farooq et al. [33]. Moreover, the potential and trends of IoT applications in the agricultural sector in Vietnam have been examined in the work by Quan et al. [34].

The potential of continuous monitoring and decision-making in sustainable agriculture, particularly concerning fertilizer usage and water consumption, has been explored by Khan et al. [35]. Soheli et al. [36] have introduced an IoT-based intelligent greenhouse monitoring system that employs wireless sensor networks (WSN) to monitor and control crucial environmental conditions such as temperature, humidity, and soil moisture. A general flowchart on how smart greenhouse works is also discussed. The collected data is transmitted to farmers through IoT and wireless communication, while the system utilizes fuzzy logic for regulating factors like heating, cooling, and humidity inside the greenhouse. Rubanga et al. [37] have developed a simplified, innovative agriculture system designed explicitly for small-scale greenhouse farming. Their approach utilizes cost-effective wireless sensor network devices and a web database for real-time data collection and monitoring of crop environments. Additionally, Kurniawan [38] has presented a prototype control and monitoring system for smart greenhouses employing fuzzy logic. Finally, Ahmed Ouammi et al. [39] have proposed a comprehensive energy management system for smart greenhouses, integrating a microgrid to optimize and control the internal environment and crop growth. These studies collectively contribute to advancing smart greenhouse technologies and utilizing IoT-based solutions in agriculture.

Numerous notable studies have delved into various aspects of smart greenhouse technology. Pereira et al. [40] proposed a framework for artificial light management in smart greenhouses, systematically investigating the impact of different light properties on plant development. Several research works have explored the combination of ventilation, horizontal airflow fans, and exhaust fans for maintaining optimal temperature and humidity levels in greenhouses [41, 42]. Ullah et al. [16] developed an optimization scheme for IoT-based smart greenhouse climate control, significantly reducing energy consumption while maintaining the desired indoor environment for maximizing crop production. Baeza et al. [43] focused on smart greenhouse covers, while Siskandar et al. [44] investigated materials and tools for assembling prototype greenhouse models. The communication among sensors in smart greenhouses is crucial, and Le et al. [45] designed a wireless sensor network based on available radio frequency technology, utilizing low-cost solar-powered units for independent sensor operation. These studies collectively contribute to advancing smart greenhouse technology and provide valuable insights into optimizing plant development, maintaining optimal environmental conditions, and establishing efficient sensor communication networks.

Lee et al. [46] conducted an assessment of irrigation efficiency for greenhouse-grown tomatoes in Lam Dong province, Vietnam. The study examined the impact of different irrigation water levels, ranging from 60% to 120% of crop evapotranspiration. Cao et al. [47] proposed an intelligent agriculture solution called iGrow, which utilizes autonomous greenhouse control. They formulated the problem as a Markov decision process optimization problem and developed a neural network-based simulator to simulate the complete planting process, enabling the optimization of control strategies. Furthermore, they introduced a closed-loop bi-level optimization algorithm that dynamically re-optimizes the greenhouse control strategy based on real-time data. Fatima et al. [48] explored the implementation of an IoT-based smart greenhouse with disease prediction using deep learning algorithms for leaf images. Several other studies have focused on integrating IoT with mobile applications for remote control of smart greenhouses [49–51]. These research efforts enhance irrigation efficiency, optimize greenhouse control strategies, and implement advanced technologies like deep learning and mobile applications in the context of intelligent greenhouse systems.

In the realm of smart greenhouse research, previous investigations have predominantly focused on studying specific aspects of the system. However, our proposed research takes a more comprehensive and holistic approach, aiming to address various interconnected components and challenges. Our study spans three years, during which we will construct an intelligent greenhouse tailored explicitly for cultivating Brassica Juncea. The extended timeframe allows us to gather actual data and insights into the optimal conditions required for the growth and development of Brassica Juncea. We aim to automate and fine-tune critical parameters influencing plant health, including temperature regulation, moisture management, plant bed optimization, fertilizer application, watering schedules, energy consumption efficiency, and ventilation roof operation. By automating these processes, we intend to create an environment that promotes optimal growth and maximizes the yield of Brassica Juncea crops. In addition to the physical infrastructure of the smart greenhouse, we propose developing a comprehensive technological solution. It includes establishing a user-friendly web interface and a mobile application that seamlessly integrates with the Internet of Things (IoT) devices deployed within the greenhouse. These technological advancements will provide real-time monitoring, control, and management capabilities, empowering farmers and researchers to remotely access and adjust various parameters to ensure optimal growth conditions for Brassica Juncea.

The web interface will be a centralized data visualization, analysis, and decision-making platform. It will allow users to monitor and assess crucial metrics such as temperature, moisture levels, nutrient availability, and energy consumption, providing valuable insights into the overall performance of the intelligent greenhouse system. On the other hand, the mobile application will offer enhanced accessibility, enabling users to conveniently control and manage greenhouse operations from anywhere at any time. By integrating IoT-based devices, our proposed system aims to streamline and automate essential tasks, reducing manual labor and improving overall operational efficiency. The synchronized control of various components, facilitated through the web interface and mobile application, will ensure that the intelligent greenhouse functions optimally, ultimately leading to increased crop productivity and yield. Through our comprehensive research approach encompassing the physical construction of the intelligent greenhouse, automation of crucial parameters, and the development of user-friendly interfaces, we aspire to contribute to the advancement of smart greenhouse technology and its practical application in the cultivation of Brassica Juncea. By leveraging the power of technology and data-driven decision-making, we aim to optimize resource utilization, enhance crop quality, and promote sustainable and efficient agricultural practices.

This paper represents a pivotal contribution to smart greenhouses and IoT research compared to previous work. Our accomplishment in this study is of paramount significance, as it marks the inception of a complete smart greenhouse IoT solution meticulously tailored for cultivating Brassica Juncea, a commonly planted mustard crop in Vietnam. Our collective accomplishments transcend the boundaries of conventional greenhouse and IoT research. Seamlessly integrating IoT technologies enables precise monitoring, data-driven decision-making, and remote management, ultimately translating into elevated crop productivity, resource efficiency, and heightened environmental sustainability within greenhouse operations. Most notably, we proudly emphasize that our work represents the pioneering effort as the first complete smart greenhouse IoT solution dedicated to the development of Brassica Juncea. This achievement underscores our commitment to advancing intelligent agriculture and highlights the novelty and uniqueness of our contribution to the field.

## 3 Construction and software design

### 3.1 Irrigation control system

The irrigation control system is a comprehensive solution encompassing various sub-modules to manage and optimize the irrigation process in a smart greenhouse. The system integrates weather, irrigation, energy, fertilizer, and personal information management to ensure efficient and effective operation [52, 53].

**Weather** The weather module records and manages essential weather parameters. It includes information such as recorded time, temperature, and humidity. This data helps monitor and adjust irrigation schedules based on current weather conditions.

**Soil Moisture** In agricultural practices, the inclusion of a soil moisture sensor is imperative. It serves as a crucial tool for determining the soil’s moisture content, helping to discern whether irrigation is necessary based on whether the soil is wet or dry.

**Beds** The bed module focuses on irrigation conditions and bed information. It records the time of irrigation, bed codes, and bed humidity. It also stores details about each bed, including the bed code, bed name, and the type of crop grown in that bed.

**Tanks** The tank module provides information about the tanks used in the irrigation system. It includes data such as tank codes, tank names, and descriptions. Furthermore, it records the water level in the tanks at different times.

**Valves and Water Pumps** The valves and water pump module manages the valves and pumps used for irrigation. It stores information about each valve, including the valve number and description. The module also records the status of valves at specific times. Similarly, it captures details about the pumps, such as pump codes, descriptions, and their respective rates.

**Crops** The crops module focuses on maintaining information related to crops. It includes crop codes, crop names, and the expected growing time for each harvest.

**Energy** The energy module provides insights into battery energy levels. It records the recording time, battery voltage, and charging mode. This information helps monitor and manage the energy resources of the system.

**Fertilizer** The fertilizer module manages information about fertilizers used in the greenhouse. It includes fertilizer codes, names, prices, inventory levels, and descriptions. Additionally, it records fertilizer schedules, such as the order number, fertilizer dates, fertilizer types, and dosages.

**User Information** The user information module stores the personal information of individuals associated with the system. It includes personal codes, full names, phone numbers, and email addresses. Furthermore, it manages account information, such as account numbers, names, and passwords.

By integrating and managing these different modules, the irrigation control system enables efficient monitoring, management, and optimization of the irrigation process in the smart greenhouse. The system provides valuable data and insights to ensure optimal irrigation conditions, effective use of resources, and streamlined operation. The irrigation control system encompasses automatic and manual operations to ensure efficient management and operation of the smart greenhouse.

**Auto Function** The auto function utilizes the parameters of weather, beds, and irrigation conditions to control valves and pumps automatically. It ensures that the system maintains optimal plant growth and development conditions. The system can adjust the irrigation schedule and water flow by continuously monitoring the weather conditions. It also optimizes the charging mode for the battery to ensure efficient energy management.

**Manual Function** In addition to the automatic function, the system provides a manual mode that allows users to intervene and control the system under certain circumstances. This manual intervention becomes necessary when the system encounters technical errors or requires maintenance. Users can manually control the following functions:

- Irrigation Management: Users can adjust the pump settings to control the water flow for irrigation. They can also manually adjust the irrigation valves to regulate the water distribution to specific beds or areas as needed.
- Power Management: The manual function allows users to adjust the charging mode of the battery manually. This feature is helpful in situations where specific power management requirements need to be addressed.

**Information management functions** Moreover, our solution supports various information management functions, such as:

- Update Fertilizer Schedule: Users can update the fertilization schedule, specifying the dates and types of fertilizers. It ensures accurate and timely application of fertilizers based on the specific needs of the crops.
- Update Fertilizer Information: Users can update information about fertilizers in the system. It includes fertilizer codes, names, prices, and inventory levels. Keeping this information up-to-date ensures accurate tracking and management of fertilizers.
- Update Personal Information: Users can update their personal information within the system. It includes personal codes, full names, phone numbers, and email addresses. Updating personal information helps maintain accurate user records and communication.
- Update Crop Information: Users can update crop information, including crop codes, crop names, and any other relevant details. Keeping crop information updated ensures accurate monitoring and management of crop-related data.
- Update Bed Information: Users can update information about the beds in the system. It includes bed codes, bed names, and any additional details related to the beds. Updating bed information helps effectively manage and monitor the irrigation process.

### 3.2 Construction of our smart greenhouse

#### 3.2.1 General requirements for constructing a smart greenhouse

Constructing an intelligent greenhouse involves careful planning and integrating various components to create an optimized environment for plant growth. Every aspect is meticulously designed to ensure efficient and sustainable cultivation, from the physical structure to the installation of sensors, automation systems, and connectivity infrastructure. Let’s delve into the key considerations and components of constructing a smart greenhouse.

**Structural Design** The greenhouse structure forms the foundation of the construction. It should be designed to provide adequate space for plant growth, allow optimal light penetration, and accommodate automation systems and equipment. Material selection, insulation, ventilation, and shading mechanisms are crucial to effectively regulating temperature, humidity, and light levels.

**Sensor Networks** Installing sensor networks is pivotal for monitoring and collecting real-time data on environmental parameters. Sensors such as temperature, humidity, light intensity, carbon dioxide levels, soil moisture, and nutrient levels are strategically placed throughout the greenhouse. These sensors transmit data to a central control system for analysis and decision-making.

**Automation Systems** Automation systems are vital in controlling and managing various aspects of the greenhouse environment. Actuators and control algorithms are integrated to automate temperature regulation, ventilation, shading, irrigation, and fertilization functions. These systems ensure precise adjustments based on sensor data, optimizing growing conditions and minimizing human intervention.

**Data Analytics and Decision-Making** The collected data from sensors is processed using data analytics techniques and algorithms. Data analysis helps identify patterns, trends, and anomalies, enabling farmers to make informed decisions. Predictive models can be developed to optimize resource allocation, manage crop growth, and detect potential issues such as pest infestation or disease outbreaks.

**Connectivity and IoT infrastructure** A robust connectivity infrastructure is established within the smart greenhouse to enable seamless communication and control. Wireless communication protocols like Wi-Fi, Bluetooth, or Zigbee connect sensors, automation systems, and the central control system. This connectivity allows remote monitoring and control, empowering farmers to access and manage greenhouse operations from any location.

**Energy Management** Smart greenhouses often incorporate renewable energy sources to power their operations. Solar panels or wind turbines can be installed to generate clean energy, reducing dependency on the grid and minimizing the environmental impact. Energy storage systems like batteries may also be incorporated to ensure a stable power supply.

**Human-Machine Interface** A user-friendly interface is implemented to facilitate interaction between farmers and the intelligent greenhouse system. This interface allows farmers to monitor sensor data, control automation systems, set parameters, and receive alerts or notifications. It can be a web-based dashboard or a dedicated mobile application, providing convenience and accessibility.

**Security and Safety** Smart greenhouses should incorporate security measures to protect the infrastructure and ensure the safety of plants. It may include surveillance systems, access control mechanisms, and fire detection and prevention systems. Proper protocols for handling chemicals, pesticides, and waste management should also be implemented to maintain a safe working environment.

In conclusion, constructing an intelligent greenhouse involves a comprehensive approach that combines structural design, sensor networks, automation systems, data analytics, connectivity infrastructure, energy management, and user interfaces. This integration enables the creation of an optimized and sustainable environment for plant growth, maximizing productivity while minimizing resource consumption and environmental impact. The authors describe our smart greenhouse construction and plant watering system in the following sub-sections.

#### 3.2.2 Brassica Juncea

Brassica Juncea, commonly known as mustard greens or gai choy, holds significant importance in the agricultural landscape of Vietnam. This leafy green vegetable is cultivated extensively and widely consumed throughout the country for its nutritional value and culinary versatility. Brassica Juncea is known for its robust growth and ability to thrive in diverse climatic conditions, making it a staple in Vietnamese cuisine. One of the notable aspects of Brassica Juncea is its exceptional nutritional profile. The vegetable offers a range of health benefits. It is a rich source of vitamins A, C, and K, crucial in supporting immune function, promoting healthy vision, and aiding blood clotting. Brassica Juncea is also known to contain significant amounts of dietary fiber, providing digestive support and contributing to overall gut health [54].

In addition to its nutritional value, Brassica Juncea plays a vital role in the agricultural landscape of Vietnam. It is a versatile crop grown year-round, offering farmers consistent income opportunities. The relatively short growth cycle of Brassica Juncea, typically 40 to 45 days, allows for multiple harvests within a single growing season, ensuring a steady supply of fresh produce. Furthermore, Brassica Juncea is highly adaptable to different climatic conditions in Vietnam, making it suitable for cultivation in various regions. It can withstand sunshine and rain, making it a reliable crop choice for farmers. Its ability to thrive in different seasons, particularly during the winter-spring crop, adds to its significance in agriculture. In Vietnamese cuisine, Brassica Juncea is a popular ingredient used in a wide range of dishes. Its distinct spicy and slightly bitter flavor adds depth and complexity to soups, stir-fries, and salads. The versatility of Brassica Juncea allows it to be incorporated into traditional dishes as well as modern culinary creations, making it a beloved ingredient among Vietnamese households and restaurants [55–57].

#### 3.2.3 Steps to construct a smart greenhouse

**Fig 1:**
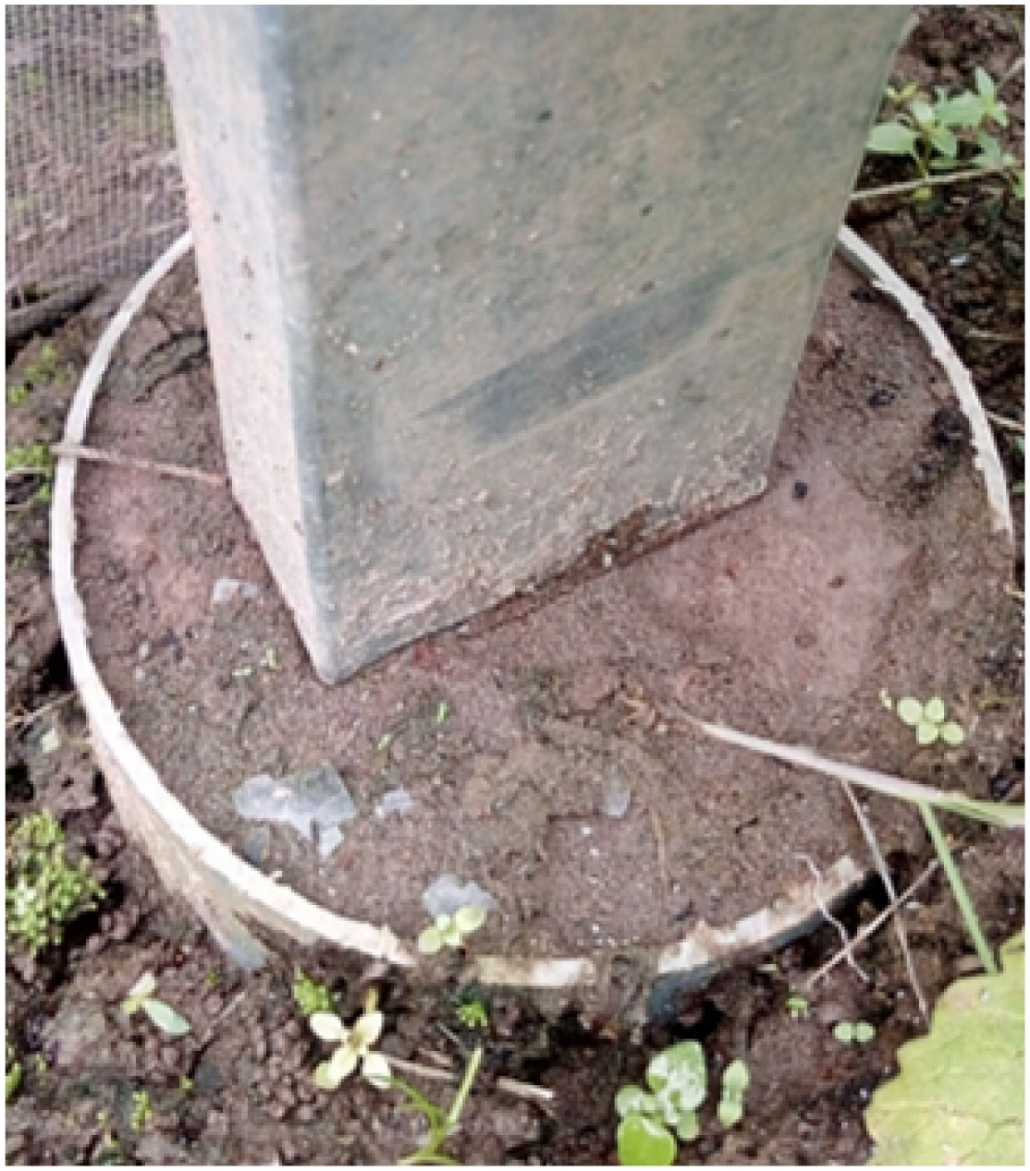
The foundation pits.

The construction of the smart greenhouse begins with the foundation pits; see Fig 1. In this case, six foundation pits are excavated and prepared to support the structure. Galvanized steel boxes with a thickness of 1.5cm are used for the foundation pits. These steel boxes offer high bearing capacity, ensuring stability for the greenhouse structure. The galvanized steel boxes are chosen to enhance the durability and corrosion resistance of the foundation pits. Galvanization involves coating the steel with a protective layer of zinc, which provides excellent anti-corrosion properties. It is essential in greenhouse environments, where moisture and exposure to elements can accelerate corrosion. Once the steel boxes are in place, concrete is poured within each foundation pit. The diameter of each pit is 0.1m, and the depth is 0.5m. The concrete provides additional stability and strength to the foundation, ensuring the greenhouse structure is securely anchored to the ground. Using concrete in the foundation pits helps distribute the load evenly and provides a solid base for the greenhouse structure. The depth of 0.5m ensures sufficient anchoring to withstand external forces such as wind or seismic activity.

**Fig 2:**
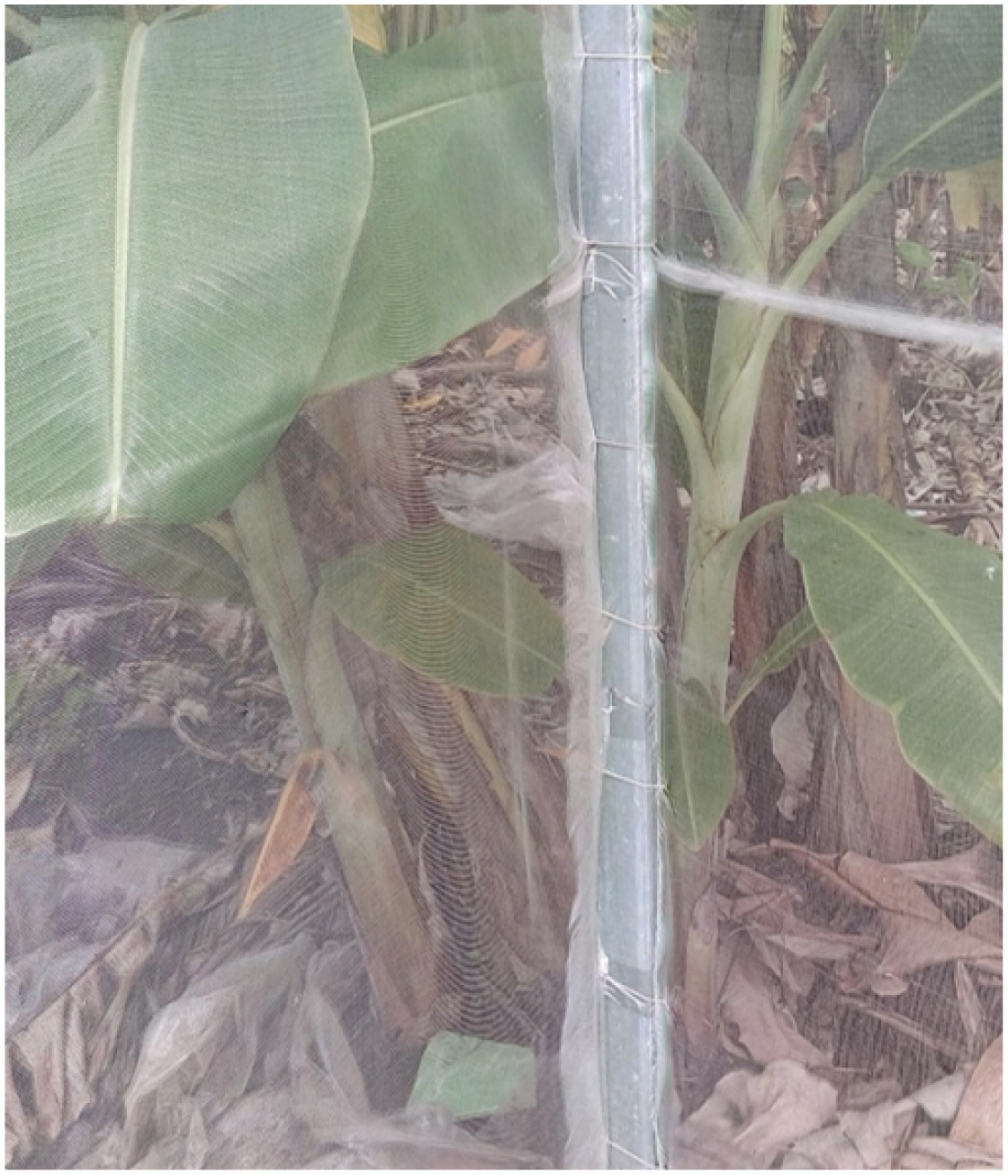
A example of a column surrounded by the insect net.

Next, the greenhouse column is designed to connect with the foundation; see Fig 2. Like the foundation pits, the greenhouse columns also utilize galvanized steel boxes with a thickness of 1.5 cm. The galvanized steel boxes provide structural strength, bearing capacity, and corrosion resistance, ensuring the durability of the columns. The column configuration consists of six columns covering an area of 30m^2^, with an approximate distance of 3m between each column. This layout helps distribute the load evenly and provides sufficient support for the greenhouse structure. To connect the foundation and the columns, 10mm bolts, and nuts are used along with temple bolts. These bolts and nuts provide a secure and stable connection between the foundation and the columns, reinforcing the overall structural integrity of the innovative greenhouse. The construction ensures a robust and reliable connection between the foundation and the columns by using galvanized steel boxes for the membrane house columns and employing appropriate connecting elements such as bolts and nuts. This design approach enhances the stability and longevity of the innovative greenhouse, enabling it to withstand external forces and maintain its structural integrity over time.

An insect net is wrapped around the structure to limit the entry of harmful insects into the smart greenhouse. The insect net is made of HDPE (High-Density Polyethylene) plastic mesh, which offers durability, strength, and resistance to environmental conditions. The insect net has a mesh thickness of 32, referring to the number of holes per inch. This density helps to effectively prevent the passage of small insects while allowing for adequate ventilation and light transmission. The net material, HDPE plastic mesh, provides several advantages. HDPE is known for its high tensile strength, making it resistant to tearing or damage. Additionally, HDPE is resistant to UV radiation, ensuring the longevity of the net even when exposed to sunlight over extended periods. The insect net has a width of 2.15m, allowing for sufficient coverage around the intelligent greenhouse. The white color of the net helps to reflect sunlight and reduce heat absorption, minimizing any potential impact on the greenhouse temperature. By utilizing an HDPE plastic mesh with a 32-mesh thickness and wrapping it around the innovative greenhouse, the insect net effectively acts as a physical barrier, limiting the entry of harmful insects. It helps to protect the plants and maintain a controlled and pest-free environment within the greenhouse, ensuring optimal growth conditions for the cultivated crops.

**Fig 3:**
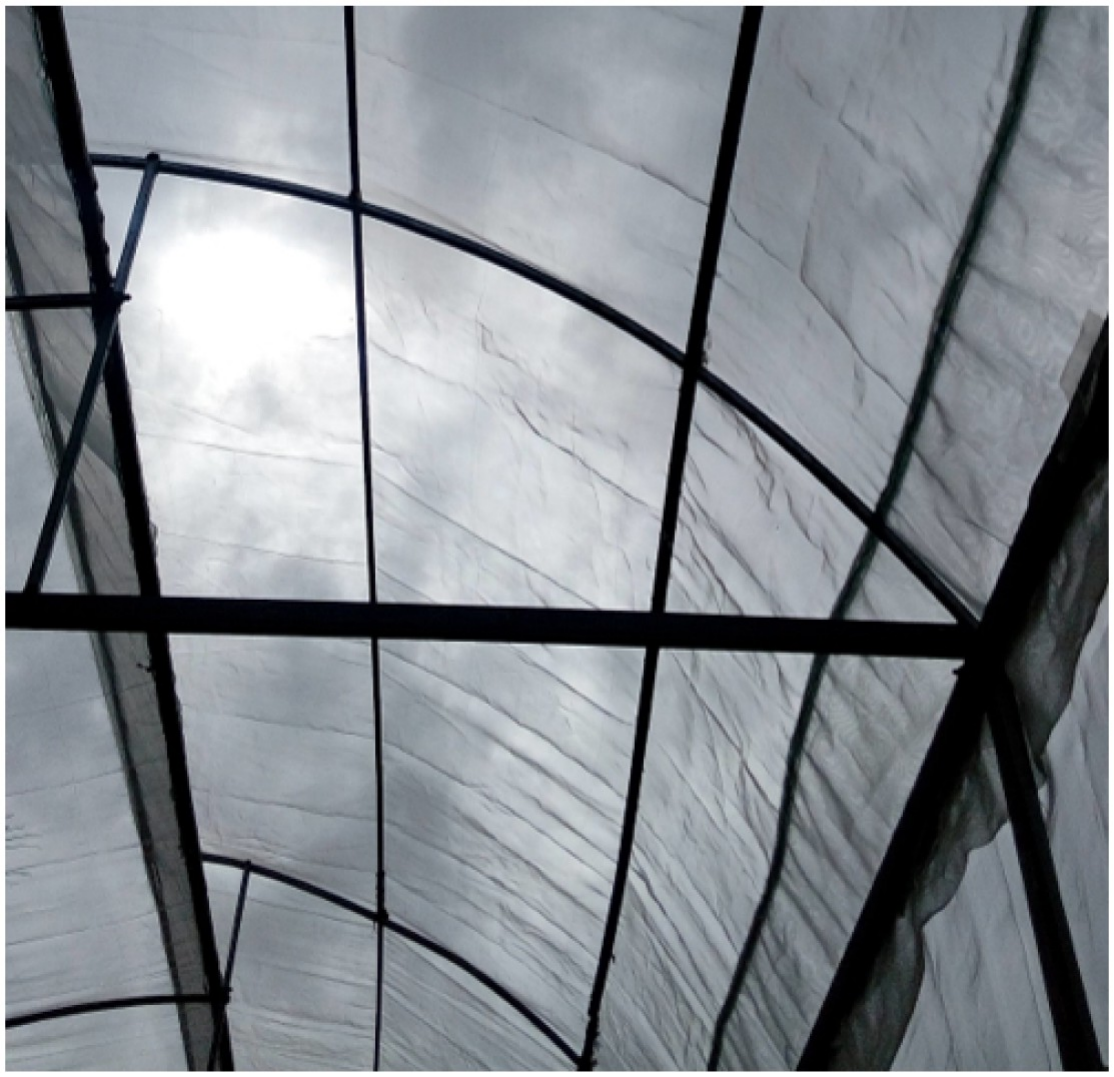
Main dome of the greenhouse.

The central dome of the greenhouse, see Fig 3, is constructed using bent galvanized box steel, which is carefully shaped into arches to form the main structure of the dome. The central arch height of the greenhouse after installation is 1.5m. This height refers to the vertical distance from the base to the arch’s highest point. The specific size may depend on factors such as the crop type, growth requirements, and available space within the greenhouse. The central dome’s arch shape allows for optimal weight distribution and provides efficient space utilization within the greenhouse. The curved design allows for better airflow and facilitates even light distribution, creating an ideal growing environment for cultivated plants.

**Fig 4:**
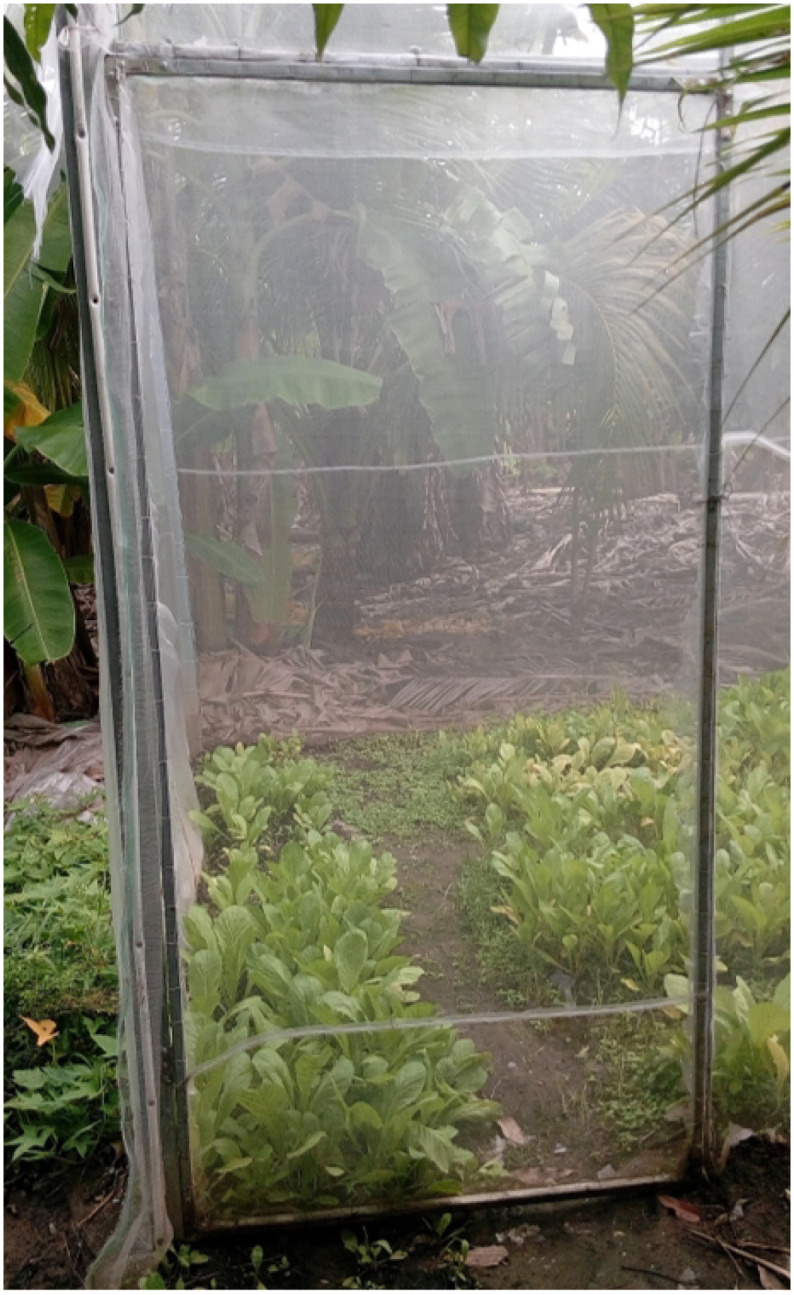
A greenhouse door.

**Fig 5:**
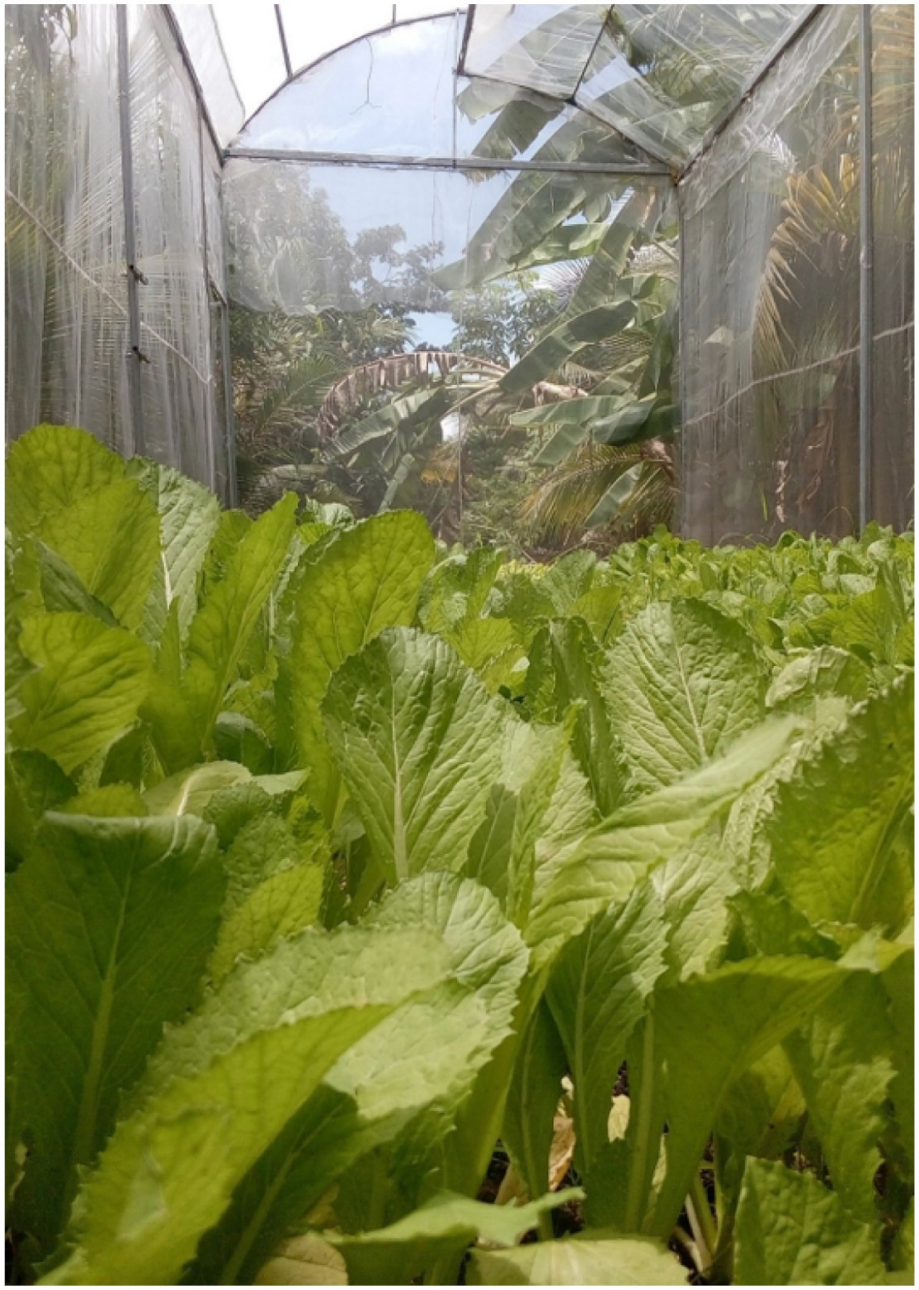
A screenshot of our smart greenhouse with Brassica Juncea in bloom.

The 2.5x2m door offers a sufficiently wide and tall opening for easy access into the membrane house. The door’s dimensions are designed to accommodate the movement of people, equipment, and materials in and out of the greenhouse. see Fig 4. The door is placed along the body of the greenhouse, strategically positioned for convenient entry and exit. The exact location of the door can be determined based on factors such as workflow efficiency, accessibility, and integration with other components of the greenhouse structure. A screenshot of our smart greenhouse and Brassica Juncea ready to harvest is presented in Fig 5.

#### 3.2.4 Essential devices for the watering system

**Fig 6:**
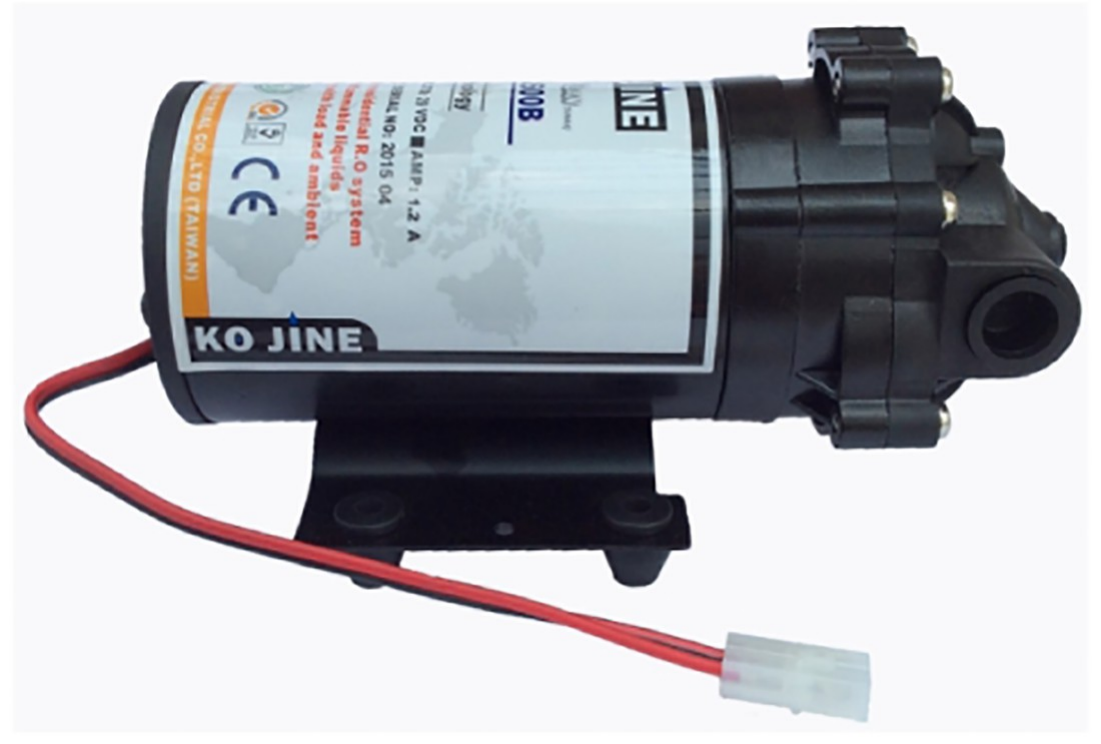
The water pump DY KJ-2600B. Image credit to bomnuocmini.com.

**Water pumps** In the irrigation system of the intelligent greenhouse, the pump plays a crucial role in various functions, such as pumping water into the water tank, misting the greenhouse for cooling purposes, and delivering fertilizer through the Venturi injector. Specifically, the DY KJ-2600B pump is utilized in this system, which operates at a voltage of 24V and a current of 230V/50HZ. The DY KJ-2600B pump, see Fig 6, is selected for its suitability and efficiency in meeting the irrigation requirements of the greenhouse. It has a pump flow rate of 1.8 L/P, indicating that it can deliver 1.8 liters of water per minute. This flow rate is designed to ensure an adequate and consistent water supply for the greenhouse’s irrigation needs. With a pressure of 130 PSI, the pump generates sufficient force to propel the water through the misting system and ensure adequate coverage within the greenhouse. The DY KJ-2600B pump has a capacity of 35W, indicating its power consumption. This information is essential for energy management and determining the overall power requirements of the innovative greenhouse.

**Fig 7:**
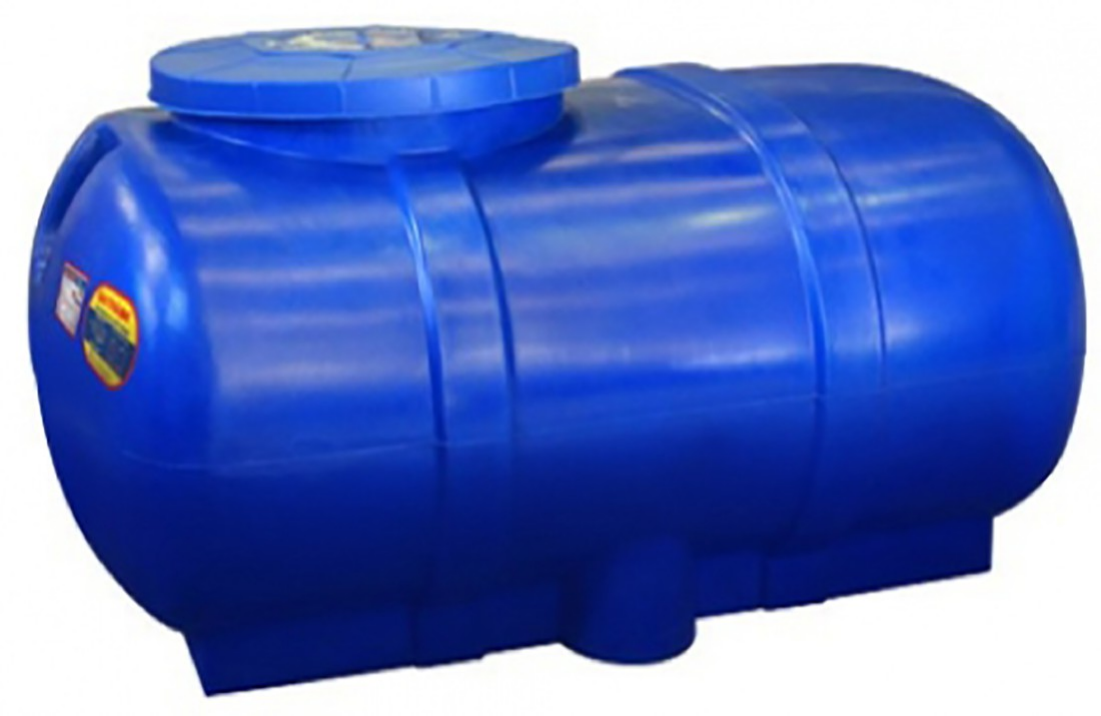
The Dai Thanh water tank. Image credit to daithanhonline.com.

**Water tank** The Dai Thanh water tank, see Fig 7, is arranged horizontally, ensuring convenient placement above the bed for easy access and efficient water distribution. It has a capacity of 300L, providing a substantial volume of water to meet the irrigation demands of the greenhouse plants. The water tank is constructed with a 4-layer thick plastic structure, which offers durability, strength, and resistance to impact. This robust construction ensures the long-lasting functionality of the tank and protects it from potential damage or degradation in the greenhouse environment.

**Fig 8:**
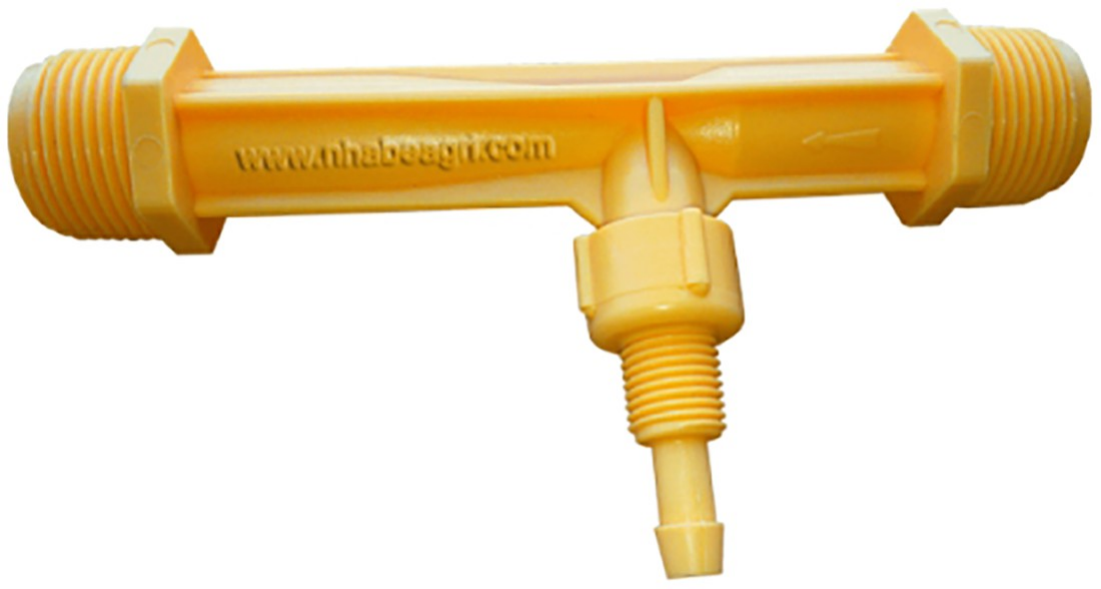
The Fertilizer parts. Image credit to www.nhabeagri.com.

**Fertilizer parts** In the intelligent greenhouse focusing on precise and standardized fertilizer application, a fertilizing system incorporating a Venturi injector, see Fig 8, is essential. The Venturi injector operates based on the principle of pressure difference created when water flows through a constricted area, generating a vacuum effect. This vacuum or suction draws fertilizers, nutrients, or other additives from tanks and containers into the irrigation system. The Venturi injector provides a reliable and efficient method of introducing fertilizers to plants within the greenhouse. As water passes through the constricted section of the injector, a pressure drop occurs, creating a vacuum or suction. This vacuum draws desired substances from separate tanks or containers such as fertilizers or nutrients. Utilizing the Venturi injector, the smart care system ensures precise control over the fertilizer applied to the plants. The suction action of the injector allows for accurate dosing and distribution of fertilizers, meeting the required standards set by organizations such as VietGab or GlobalGab. It helps to maintain optimal nutrient levels in the greenhouse, promoting healthy plant growth and maximizing crop yields. Additionally, the Venturi injector provides versatility regarding the substances that can be introduced into the irrigation system. It enables the injection of various additives, including fertilizers, nutrients, or even pH adjusters, depending on the plants’ specific needs.

**Fig 9:**
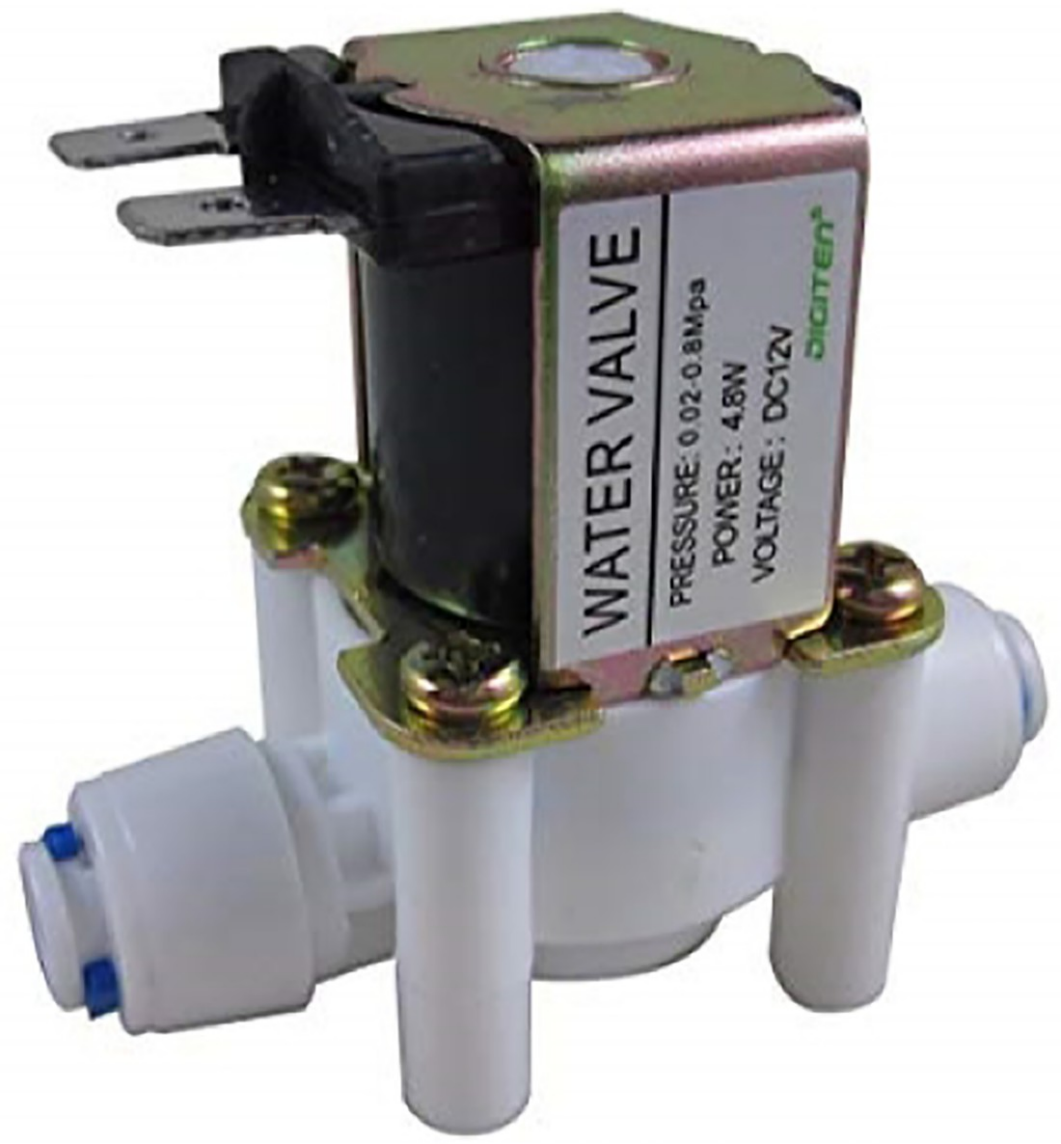
The electromagnetic valve. Image credit to bomnuocmini.com.

**Electromagnetic valve** The electromagnetic valve, see Fig 9, is a critical component in the intelligent greenhouse system responsible for controlling liquid flow through the system. It has several key features, including an inductor, iron core, compression spring, and rubber gasket. Under normal circumstances, without electricity, the compression spring exerts pressure on the iron core, keeping the valve closed. However, when an electric current passes through the inductor, it generates a magnetic field that attracts the iron core, pulling it out against the force of the spring. This magnetic force is strong enough to overcome the spring tension, allowing the valve to be open and liquid to flow through the system. The valve used in the system is designed for a pipe diameter of 0.5 inches. It operates with a voltage of 12VDC, which is suitable for compatibility with the intelligent greenhouse’s electrical system. The electromagnetic valve can handle liquid flow with a 0.2 to 0.8 Mpa pressure range. This flexibility in pressure handling ensures that the valve can adapt to different flow rates and pressure conditions within the greenhouse system. Regarding response time, the solenoid valve has a quick opening time of less than or equal to 0.15s, allowing for swift initiation of liquid flow when required. When closing the valve, the response time is also relatively fast, less than or equal to 0.3s, ensuring efficient control and precise management of liquid flow. The valve is designed to operate within a temperature range of 1 to 75°C. This temperature tolerance allows the valve to function effectively in various environmental conditions, ensuring its reliability and durability within the intelligent greenhouse system.

**Fig 10:**
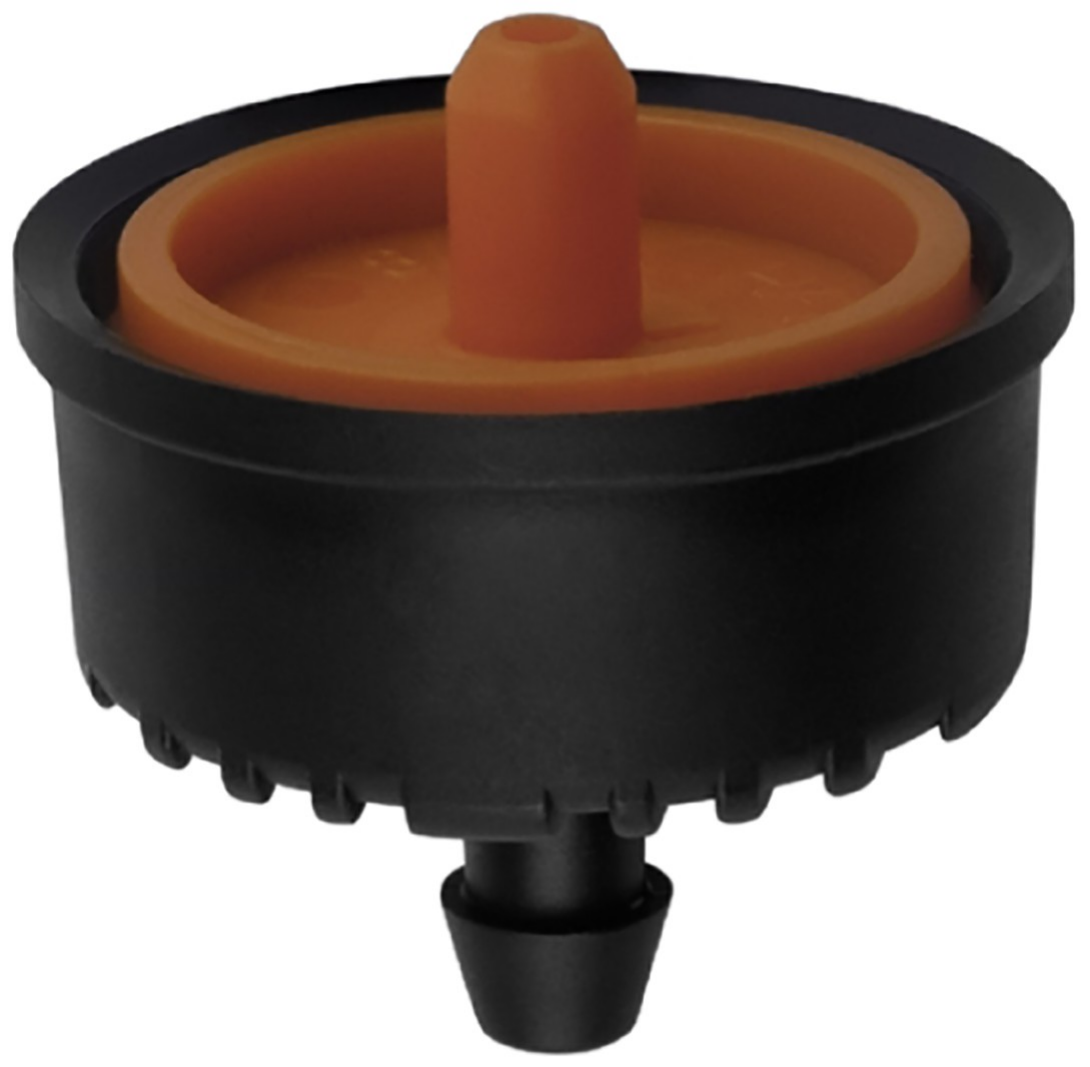
A drip irrigation head. Image credit to www.nhabeagri.com.

**Drip irrigation head** The pressure compensating drip irrigation heads, see Fig 10, are designed to maintain a consistent flow rate, regardless of variations in water pressure within the pipeline system. It ensures that each crop receives equal water, nutrients, and fertilizers, promoting consistent growth and preventing over- or under-irrigation. The specific drip irrigation heads chosen for the intelligent greenhouse system have a flow rate of 3L/H. This flow rate indicates that each irrigation head delivers 3 liters of water per hour to the plants. The flow rate is carefully selected based on the cultivated crops’ water requirements. The smart greenhouse system achieves precise and controlled irrigation by utilizing pressure-compensating drip irrigation heads with a flow rate of 3L/H. It supports optimal plant growth and helps conserve water and maintains efficient resource usage.

**Fig 11:**
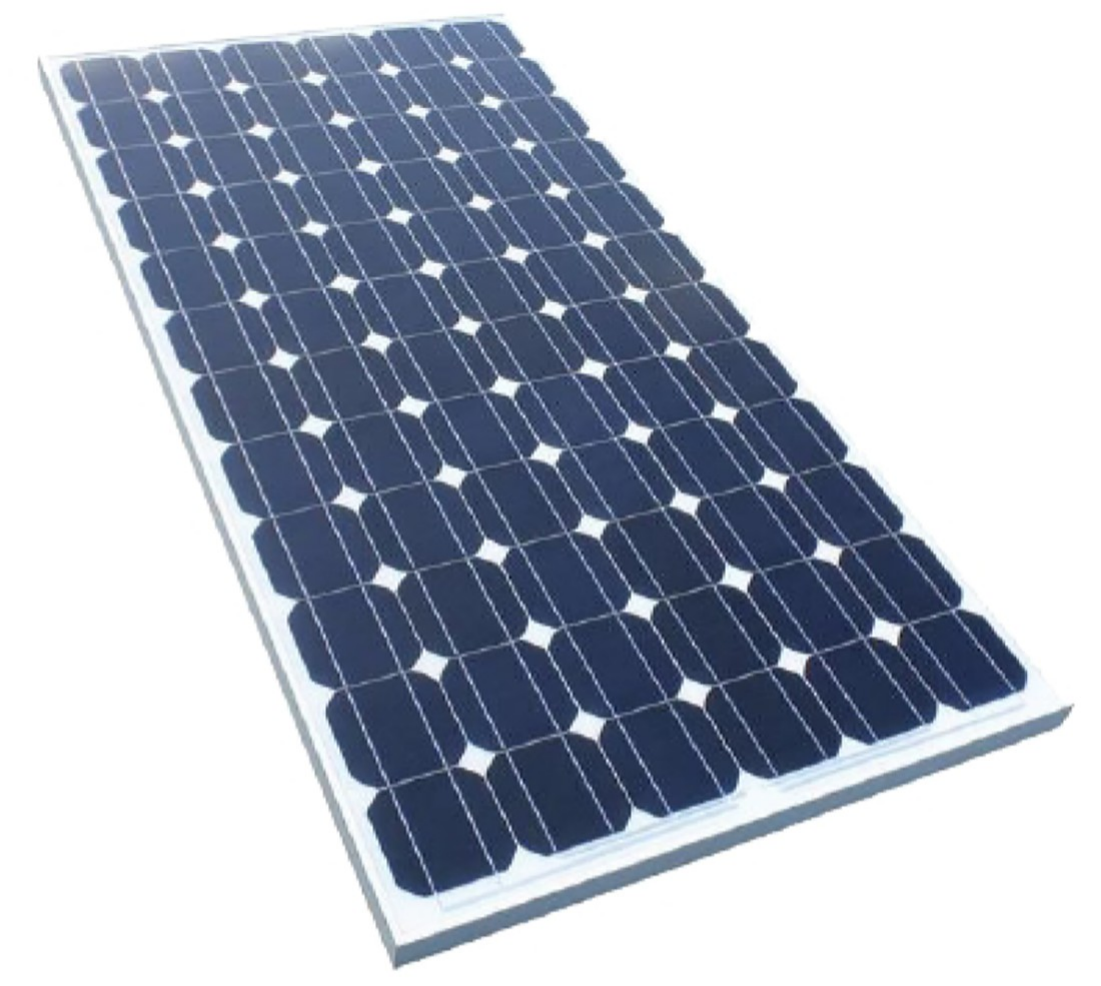
A Solar panel. Image credit to solarstore.vn.

**Solar panels** A solar panel is indeed a device that converts sunlight directly into electrical energy through the photovoltaic effect; see Fig 11. In the context of our greenhouse construction, solar panels are utilized to harness solar energy and generate electricity to power various devices and systems. Solar panels are typically composed of interconnected solar cells responsible for converting sunlight into usable electrical energy. The use of solar panels in the intelligent greenhouse offers several advantages. Firstly, it provides a clean and renewable energy source, reducing reliance on traditional fossil fuel-based electricity. Secondly, solar panels can operate silently and require minimal maintenance, making them a reliable and cost-effective energy solution. Additionally, solar panels contribute to the overall sustainability and eco-friendliness of the greenhouse by reducing carbon emissions and environmental impact.

**Fig 12:**
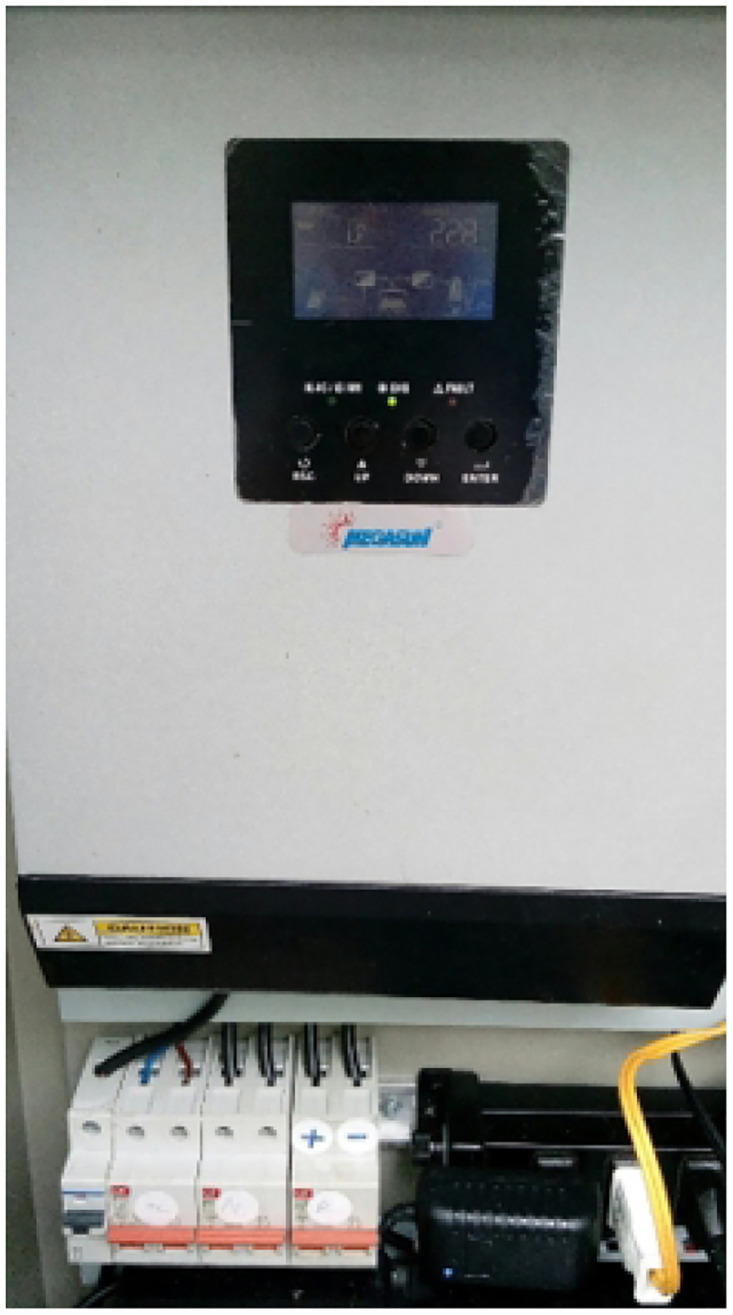
An universal DC-AC converter. Image credit to www.megasun.com.vn.

**Universal DC-AC Converter** The universal DC-AC converter, see Fig 12, is a versatile inverter series that integrates multiple functions, including an inverter, solar charger, and battery charger. This device offers flexibility and convenience in managing the power supply within the intelligent greenhouse. The converter features an LCD configuration interface, allowing easy setup and customization of settings. Users can navigate the menu and adjust according to their specific requirements using the function keys on the inverter. Keeping the battery close to the charger is recommended to ensure optimal performance and safety, and it helps minimize power loss during charging and provides efficient energy transfer. Additionally, selecting high-quality wires for the installation is advisable, as poor-quality wiring can lead to power losses and inefficiencies. It is essential to avoid exposing the battery and charger to direct sunlight. Direct sunlight can increase the temperature of the components and adversely affect their performance and lifespan. Therefore, installing the converter in a well-ventilated area is preferable to dissipate any heat generated during operation. The charger is designed to automatically recognize the battery voltage, distinguishing between 12V and 24V systems. During the initial installation, it is crucial to ensure that the battery is fully charged to enable accurate voltage recognition by the converter. It is essential to note that the charger is specifically designed for use with 12V/24V OPEN, AGM, and GEL lead-acid batteries. It should not be used with lithium batteries or other battery types, as this can damage the circuitry or cause other operational issues. Furthermore, using the charger exclusively with solar batteries and avoiding connecting it to other DC input sources is essential. Misusing the charger or with incompatible input, sources can lead to circuit malfunctions or even damage the device. By adhering to these guidelines and recommendations, the universal DC-AC converter can operate efficiently, deliver reliable power conversion, and contribute to the intelligent greenhouse system’s overall functionality and energy management.

**Fig 13:**
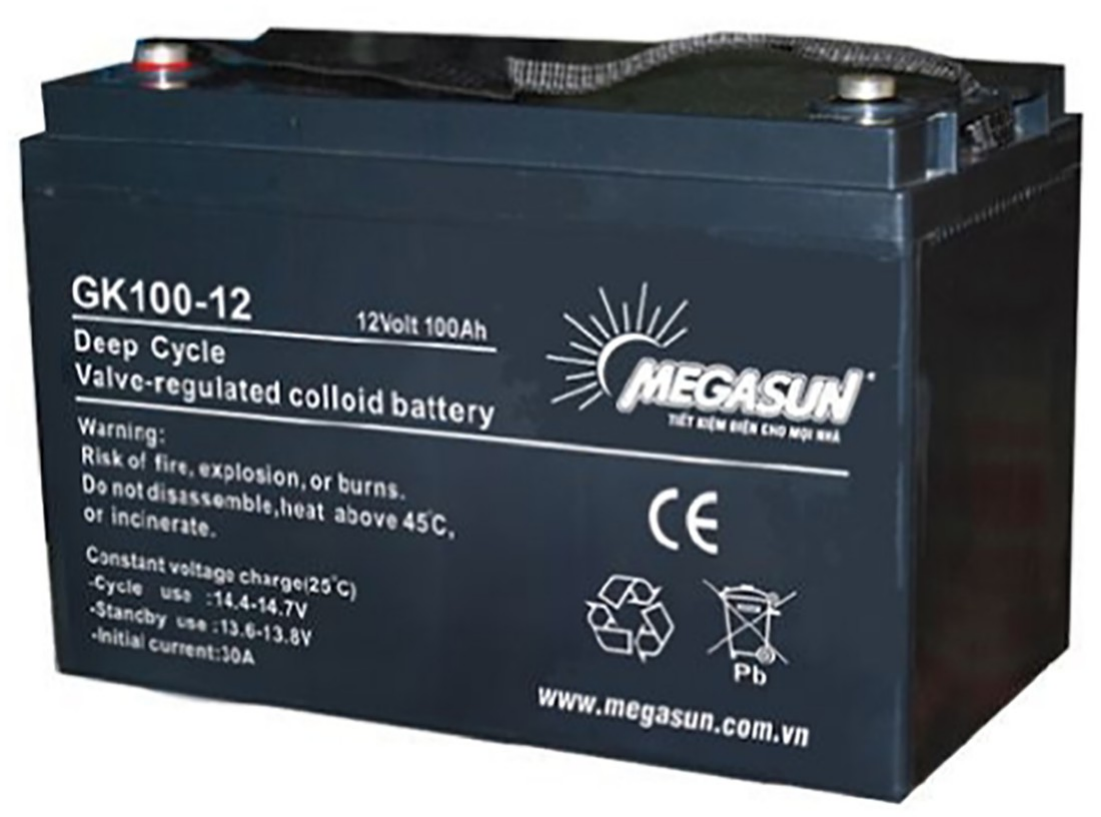
A deep discharge battery. Image credit to www.megasun.com.vn.

**Deep discharge battery** A deep discharge battery, see Fig 13, also known as a deep cycle battery, is specifically designed for use in solar energy systems to store and provide energy. It is built to endure repetitive deep discharge and charge cycles, making it suitable for sustained and reliable operation. Their large capacity and heavy-duty construction characterize deep-discharge batteries. They are designed to handle the demands of renewable energy systems, where frequent charging and discharging are standard. Unlike regular batteries, deep discharge batteries are constructed with thicker and more robust plates, enabling them to withstand the stress of deep cycling. Deep cycling is the process of discharging a battery to a significant extent before recharging it, and it contrasts with shallow cycling, where batteries are only partially discharged before recharging. Deep cycle batteries are optimized for deep cycling, allowing them to provide a stable and reliable power supply over an extended period. In solar energy systems, deep discharge batteries play a crucial role in storing excess energy generated by solar panels during periods of high sunlight. Its kept energy can then be utilized during periods of low sunlight or increased energy demand. Deep cycle batteries enable efficient energy management within the system, ensuring a consistent power supply for devices and equipment. It is important to note that deep discharge batteries do not typically feature an inner foam sheet. This design choice enhances their durability and resilience to deep cycling, as the absence of the foam sheet allows for better structural integrity and improved performance under repeated charge and discharge cycles.

**Fig 14:**
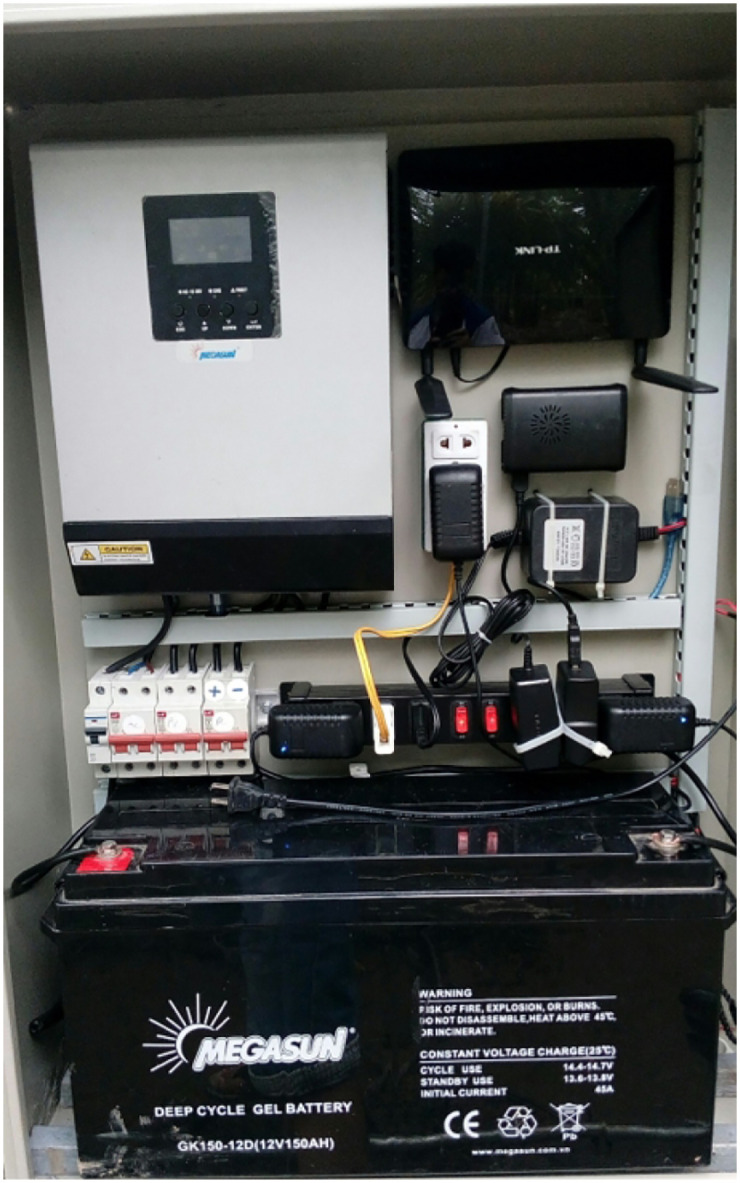
The central control box.

**Fig 15:**
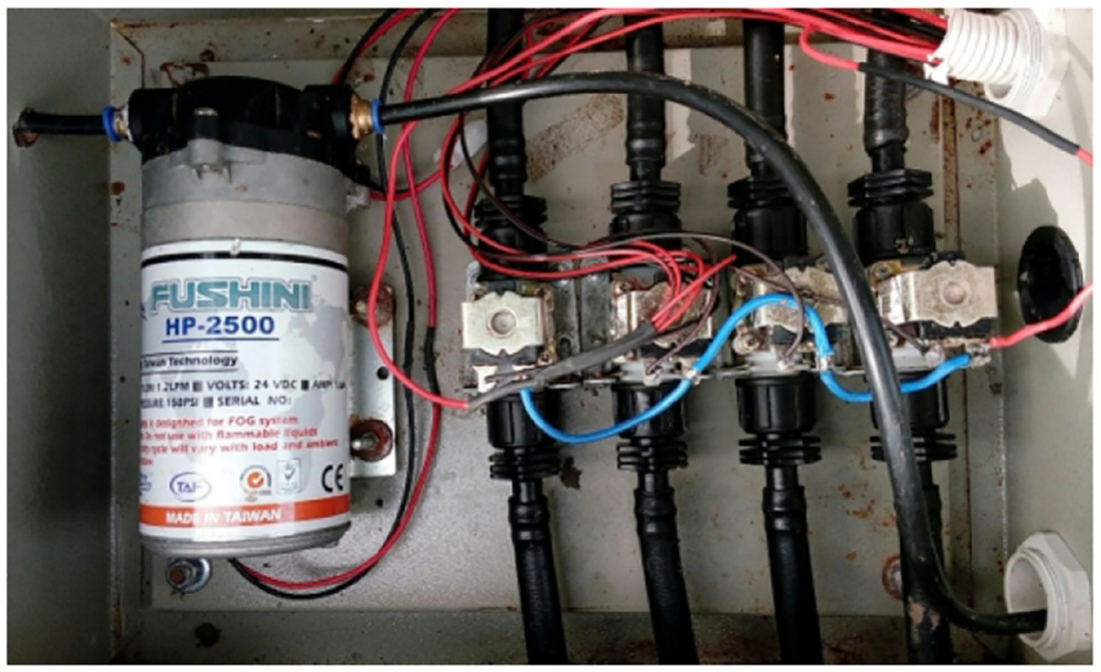
The irrigation box with a water pump.

**Fig 16:**
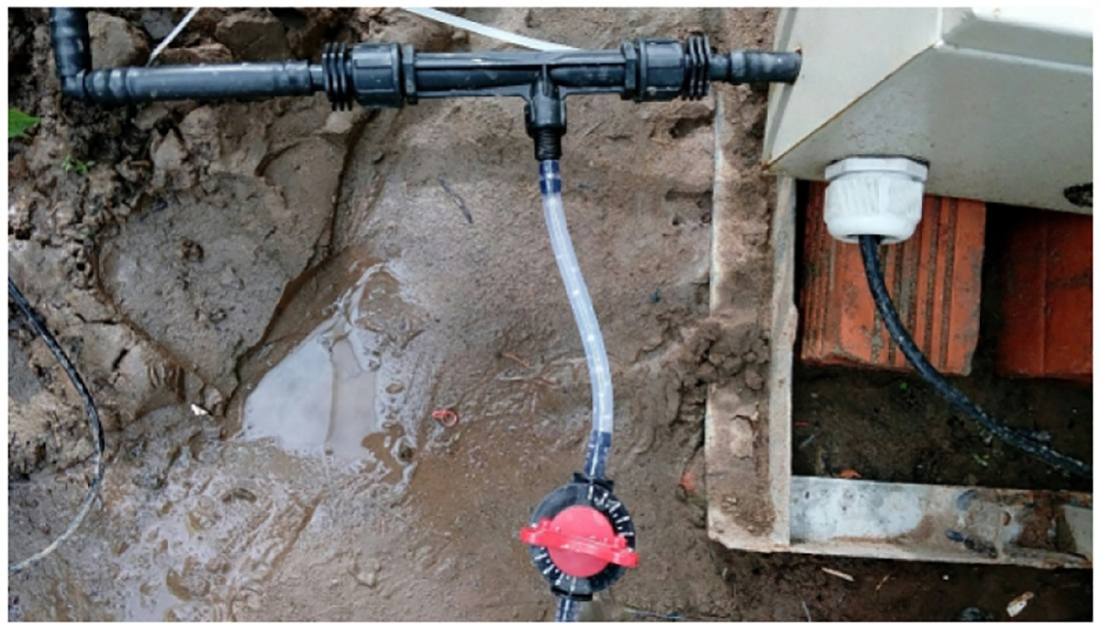
Joint connection to fertilizer containers.

**Fig 17:**
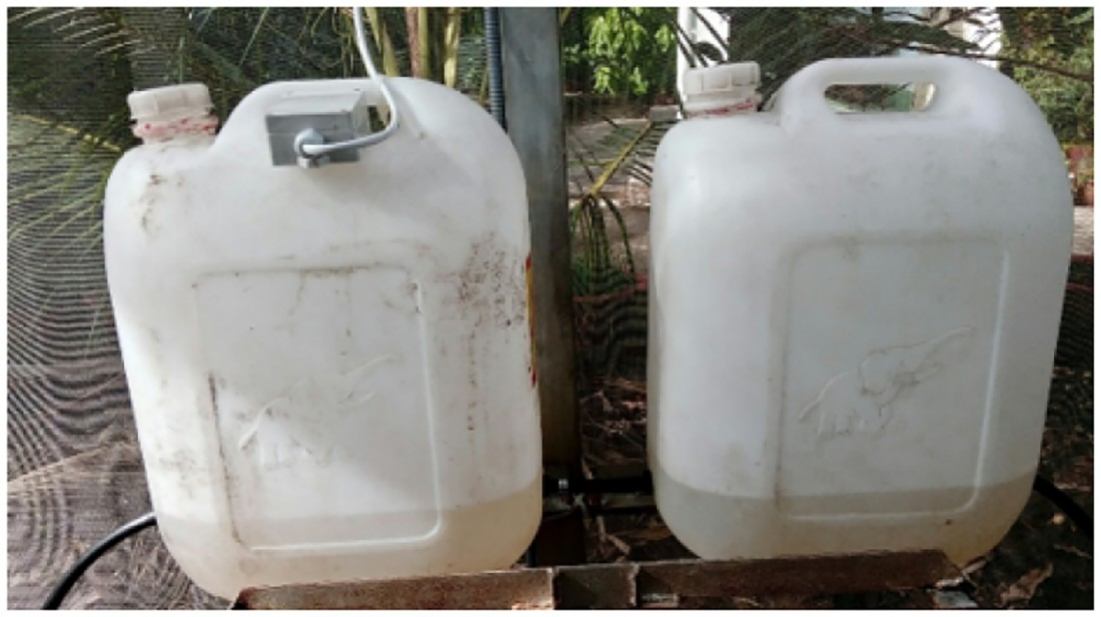
Fertilizer containers.

**Fig 18:**
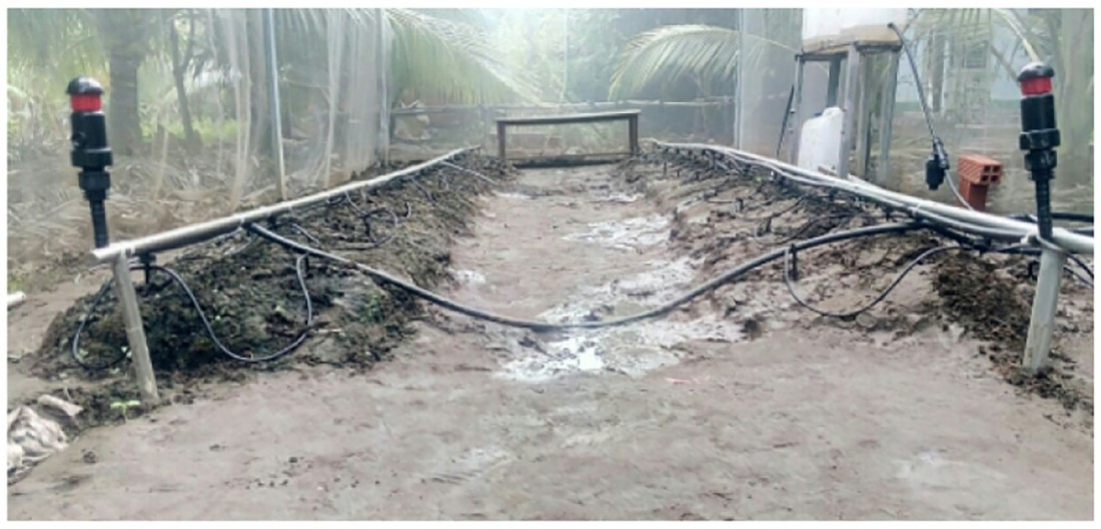
Two irrigation pipes in the greenhouse.

A control box is a centralized unit that houses and organizes various devices and components in one location. It serves as a hub for controlling and managing the connected devices effectively. We show a box in Fig 14. In addition, several screenshots of our irrigation system are presented in Figs 15–18.

### 3.3 Internet of things

The Internet of Things (IoT) is a concept where every object and individual is assigned a unique identifier, enabling them to transmit and exchange information and data through a network. This network operates independently, without direct human-to-human or human-to-computer interaction. The development of IoT has emerged from the convergence of wireless technology, microelectronics, and the Internet. It comprises various devices capable of connecting to the Internet and interacting with the external world to perform specific tasks [58]. Extensive research on IoT and environmental sensors provides a solid foundation for the advancement of monitoring and control systems in smart greenhouses [36, 40, 59, 60].

Connections within the IoT can be established using technologies such as Wi-Fi, broadband telecommunications networks (xG), Bluetooth, ZigBee, and infrared. These devices include smartphones, coffee makers, washing machines, headphones, light bulbs, and more. According to the International Data Corporation (IDC), it is projected that by 2025, there will be approximately 41.6 billion IoT devices, potentially generating an enormous amount of data, estimated at 79.4 zettabytes (ZB) [61]. IoT is envisioned as a vast network that interconnects everything, including people, fostering relationships between people and devices.

An IoT network can accommodate an astonishing number of connected objects, ranging from 50 to 100 trillion, with the capability to track the movement of each object. In urban settings, individuals may find themselves surrounded by anywhere from 1000 to 5000 things equipped with tracking abilities. For an IoT system to be considered adequate, it must meet the following requirements:

**Connection based on identity** “Things” must have a unique ID. The IoT system needs to support connections between “Things” and the connection is established based on the identifier (ID) of the Things.

**Interoperability** IoT systems interoperability between networks and Things.

**Self-governance of the network** Includes self-management, self-configuration, self-healing, self-optimization, and self-protection. The network must adapt to different application domains, communication environments, and devices.

**Agreement service** This service can be provided by automatic collection, communication, and data processing between “Things” based on rules established by the operator or customized by the user.

**Location-based capabilities** Communication and services related to something depend on the location information of Things and the user. The IoT system can know and track the location automatically. Law or regulation may restrict location-based services and are subject to security requirements.

**Security** In IoT, many “Things” are connected. Sometimes, it increases security risks, such as disclosing confidential information, incorrect authentication, or altering or tampering with data.

**Privacy protection** All “Things” have their owners and users. Data collected from the “Things” may contain personal information regarding its owner or user. IoT systems must protect privacy during data transmission, aggregation, storage, mining, and processing, and protecting privacy should not establish a barrier to data source authentication.

**Plug and play** Things must be plug-and-play easily and conveniently.

**Manageability** The IoT system needs to support the management of “Things” to ensure the network works properly. IoT applications often work automatically without human involvement, but the stakeholders should manage their entire operation.

### 3.4 Software framework

#### 3.4.1 Web services

Web Service is a set of open protocols and standards to exchange data between applications or systems. Web Service is an integration between two computers, enabling two computers to interact with each other over the network effectively. Web Service allows a computer program to *talk* to a website instead of the user having to browse to access the website manually [62, 63]. Software applications are often written in programming languages or run on different platforms. In addition, they can use Web Service to exchange data and communicate between processes on a computer. Web services are divided into two categories: SOAP and RESTful.

SOAP Web Service is “Simple Object Access Protocol Web Service” [64]. It is considered one of the protocols built on XML to define plain-text data over HTTP. SOAP Web Service in Java is considered a solid pillar for distributed applications as the framework comprises many languages and operating systems. Web Services often use SOAP to transmit data. Because it is based on XML, SOAP Web Service is considered a protocol independent of the platform or any programming language. Users can write SOAP in Java, PHP, and .NET or deploy it on Windows and Linux.

REST stands for REpresentational State Transfer. REST is seen as a software architecture, not a protocol [65]. And RESTful Web Services are understood as Web Services have written based on REST architectures. Besides, REST is widely used as an alternative to SOAP and WSDL-based Web Services. Like SOAP, RESTful Web Service does not depend on any platform or programming language. Therefore, REST can use SOAP Web Service as an implementation of REST. In recent years, regarding the service design model, REST has been highly appreciated and is almost seen as able to replace both SOAP and WSDL. REST defines architectural rules that help users design Web Servers, and it focuses on the resource system and is written in many different languages. The basic principles of REST in design: display directory structure as URLs; REST uses a very explicit HTTP method; stateless; and REST conveys JavaScript Object Notation, XML, or both.

Today, along with the development of the Internet, Web Service has also become a technique used to link and interact between applications on different computers through the Internet environment. More service providers want to bring services to the public, and the biggest problem that providers are facing is the security of Web Services [66]. Securing Web Services is a fundamental issue, especially for finance, the stock market, and e-commerce services. The problem is how to securely exchange information and data without being attacked. Web Service Security is a security standard for SOAP and SOAP extensions. It is used when you want to build secure and reliable web services, and web Service Security guarantees message integrity and reliability. The server and the client need to implement some security regulations to create information security in the application.

**Client-side** Specifies the components of the message that must be signed or otherwise authenticated. Specifies a file system key that will mark the message and that only authorized machines own this key. Specifies the algorithms that will be used by the key to sign the message.

**Server-side** Specifies which message components need to be signed. The request fails if the incoming message has a valid signature. Specifies a key to validate the signature of the incoming message. Specifies the proper decryption that the key uses to ensure the integrity of the incoming message. The response message must be signed, and provide signature information when responding.

#### 3.4.2 PHP

Hypertext Preprocessor (PHP) is a scripting language or code primarily used to develop server-written, open-source, general-purpose applications. It is very web-friendly and can be easily embedded into HTML pages [67–69]. Because it is optimized for web applications, is fast, compact, has a syntax similar to C and Java, is easy to learn, and has a relatively shorter product build time than other languages, PHP has quickly become one of the most popular programming languages in the world. Installing and using PHP is very easy and free. The PHP community is quite large and of good quality, and the applicability is very high. With a large development community, updating new versions, current version errors, and testing new versions make PHP very flexible in improving itself. In addition to the community’s support, the PHP script library is prosperous and diverse, from tiny things like just a piece of code, a function (PHP.net) to bigger codebases like Frameworks (Zend, CakePHP, CodeIgniter, Symfony, Laravel) or complete application (Joomla, WordPress, and PhpBB). Since PHP 5 version, PHP has supported most of the outstanding features of object-oriented programming, such as Inheritance, Abstraction, Encapsulation, Polymorphism, Interface, and Autoload. With more and more PHP frameworks and applications written in the OOP model, developers can easily and quickly access and extend these applications.

#### 3.4.3 Laravel framework

Developed based on the Model-View-Controller model, Laravel is a free and open-source PHP Framework with clear, coherent syntax. Model-View-Controller (MVC) is a software architectural pattern that is widely used in web development. It separates an application’s data (the model), user interface (the view), and control flow (the controller) into separate components. The model represents the data and business logic of the application, the view displays the data to the user, and the controller handles user input and updates the model and view accordingly. By separating these concerns, the MVC pattern promotes a modular and flexible design that is easier to maintain, test, and extend [70–72].

**Model** This is where the business interacts with data or the database management system (MySQL). The Model component includes classes/functions that handle many operations such as database connection, data query, and add—delete—edit data.

**View** This is where interfaces such as buttons, input frames, menus, and images are stored. The View component will be responsible for displaying data and helping users interact with the system.

**Controller** This is the place to receive processing requests sent from users. The Controller component will include classes/functions that handle many business logic to help get the correct data and information and display that data to the user through the View class.

#### 3.4.4 Android

An Android application is a software program developed to run on the Android operating system. It consists of two main components: the source code, which is written in Java, and the user interface (UI) designed using XML markup language [73–75]. XML, or Extensible Markup Language, is a versatile markup language used to encode data in a format that is both human-readable and machine-readable. Initially introduced in the late 1990s, XML aimed to standardize data representation in web applications. Over time, XML has gained widespread adoption across various domains, serving purposes such as data storage, data interchange, and configuration files. XML documents are composed of elements and attributes, defining the structure and content of the encoded data. XML’s extensibility enables developers to define custom tags and attributes, allowing for the creation of new elements and expanding the language’s capabilities. The human-readable and machine-readable nature of XML makes it an ideal format for exchanging information between different software systems and platforms. The user interface in an Android application is designed based on programming principles, while the interaction with the interface is implemented in the source code. The UI serves as the visual representation of the application, allowing users to interact with its features and functionalities. From a programming perspective, Android applications are composed of various components and are categorized into six main categories. These categories encompass activities, services, broadcast receivers, content providers, fragments, and intents. Each component serves a specific purpose within the application and contributes to its overall functionality and user experience.

**Activity** An Activity is an application component that provides a screen for users to interact to do something. Similar to *main()* method in many programming languages, the Android system initiates code in an Activity instance. Main Activity is the first screen to appear when a user launches the app.

**Service** As a component that runs in the background, the main function of this component is to update data, give notifications and not frequently notify the user.

**Content Provider** Content provider is a shared data store. The main function of this component is to manage and share data between applications.

**Intent** Considered as the soul in Android applications, the function of Intent is to transmit messages and data between Activities to initialize Activity or Services.

**Broadcast Receiver** A component that receives Intents from outside the application. For example, the program gets messages and receives calls.

**Notification** Issue alerts without causing the Activity to be down.

Each Android application has different process states. The programmer must understand these states of the application because low-priority processes can be released without warning. In general, the application has the following process states:

**Foreground Process** The process of the current application being interacted with by the user.

**Visible Process** The application process that the Activity shows to the user.

**Service Process** The Service is running.

**Background Process** The application process whose activities are not visible to the user. *onStoped()* of the Activity is called.

**Empty Process** Process does not have any active components.

Each Android application usually has many *Activity*, and each Activity has a separate life cycle, which is divided into the following phases:

**Entire lifetime** From *onCreate()* to *onDestroy()* methods.

**Visible liftetime** From *onStart()* to *onStop()* methods.

**Foreground lifetime** From *onResume()* to *onPause()* methods.

At each different stage in the lifecycle, an Activity will have other states:

**Active (running)** Activity is displayed on the screen (foreground).

**Paused** Activity is still visible but cannot be interacted with (lost focus).

**Stopped** When another Activity entirely replaces the Activity, it will enter the Stopped state.

**Killed** When memory is short, the system will release Processes according to the priority principle. The empty process will be released first. Activities in Paused or Stopped state can also be released.

When building an Activity for an application, the *onCreate()* method is constantly rewritten to perform initialization. Other methods can be rewritten or not depending on the requirements of the problem to be solved.

Android Studio is the official IDE used in Android application development based on IntelliJ IDEA. The primary function of Android Studio is to provide interfaces that allow users to create applications and handle complex file tools behind the scenes. The programming language used in Android Studio is Java, which will be pre-installed on your device. When using Android Studio, you need to write, edit and store them on your projects and the files within that project.

### 3.5 Essential hardware and IoT devices

#### 3.5.1 Ultrasonic distance sensor HC-SR04

**Fig 19:**
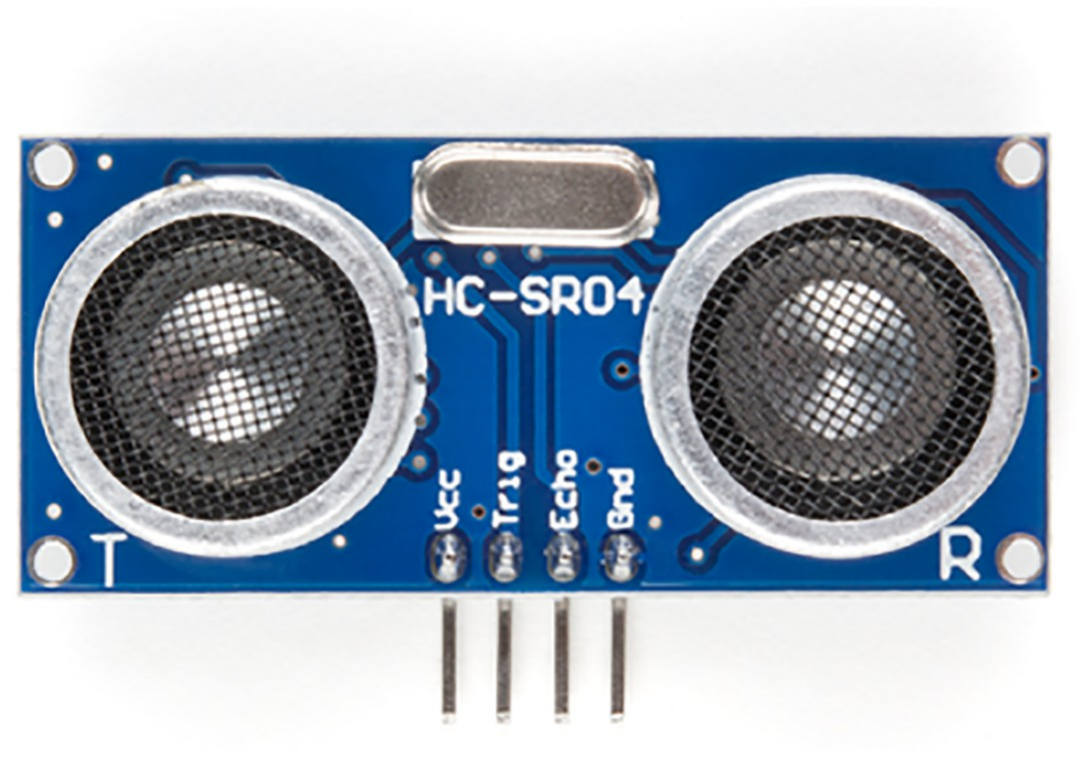
HC-SR04 sensor. Image credit to www.sparkfun.com.

The HC-SR04, see Fig 19, is a cost-effective sensor offering a non-contact measurement capability ranging from 2cm to 400cm, with an impressive accuracy of up to 3mm. Each HC-SR04 module consists of an ultrasonic transmitter, a receiver, and a control circuit. When it comes to connecting the HC-SR04, you only need to worry about four pins: VCC (Power), Trig (Trigger), Echo (Receive), and GND (Ground). Setting up and utilizing this sensor for your range-finding projects is a breeze! What sets this sensor apart is its built-in control circuitry, which effectively minimizes inconsistent and erratic data, especially in situations where bouncing measurements may occur. The HC-SR04 sensor works best between 2 cm—400 cm within a 30-degree cone and is accurate to the nearest 0.3cm. Table 1 lists the HC-SR04’s specifications.

**Table 1:** Technical specifications of the HC-SR04 sensor.

Input voltage	5V
Current draw	max 20mA
Digital output	0V to 5V
Working temperature	-15° to 70°C
Sensing angle	30° cone
Angle of effect	15° cone
Resolution	0.3cm
Ultrasonic frequency	40kHz
Range	2cm to 400cm
Dimension	45mm x 20mm x 15mm

#### 3.5.2 Relay soil moisture sensor HT195

**Fig 20:**
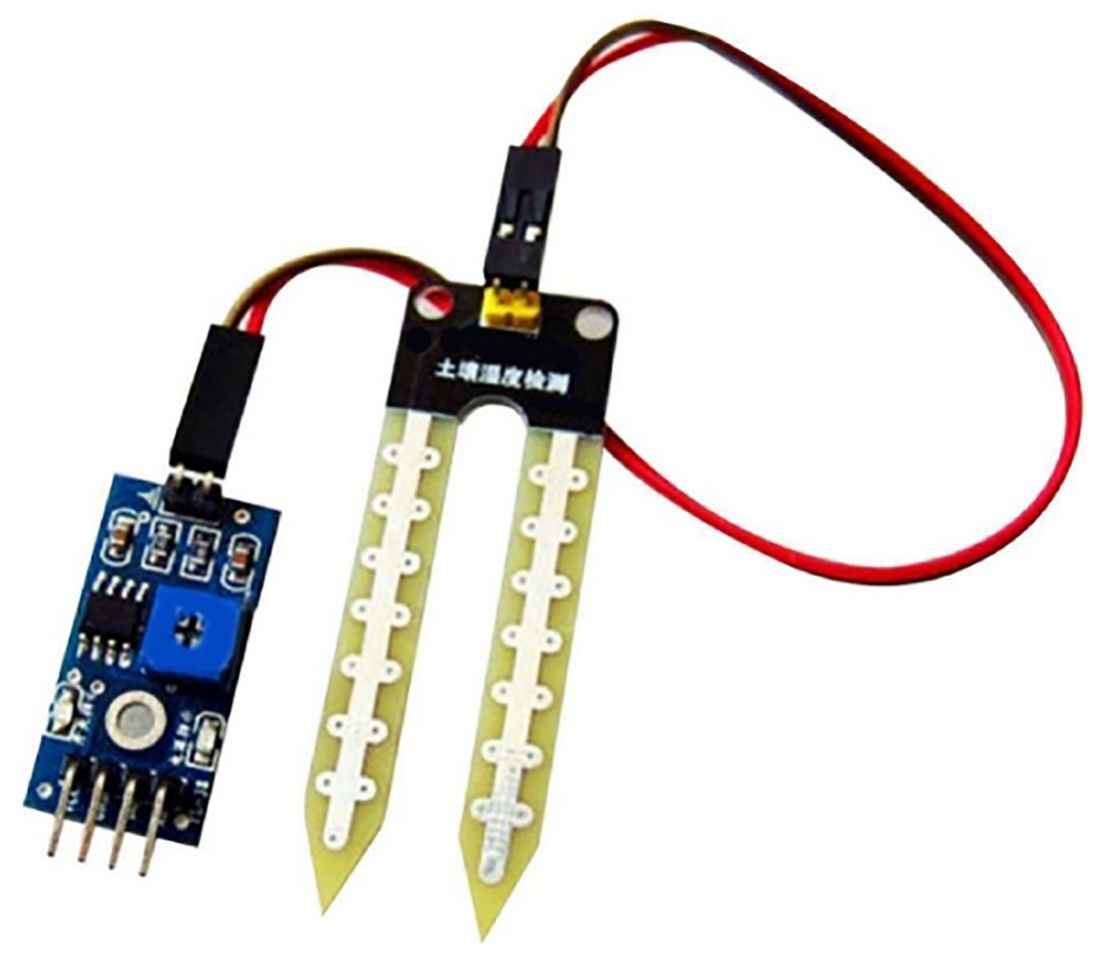
HT195 module. Image credit to www.amazon.com.

The HT195 module, see Fig 20, consists of a moisture sensor, resistors, capacitor, potentiometer, comparator LM393 IC, power, and status LED in an integrated circuit. It is used to detect the soil’s moisture, and it measures the volumetric content of water inside the soil and gives us the moisture level as output. The module has digital and analog outputs and a potentiometer to adjust the threshold level. Table 2 lists the HT195’s specifications.

**Table 2:** Technical specifications of the HT195 sensor.

Operating voltage	3.3V to 5V DC
Operating current	15mA
Digital output	0V to 5V, adjustable trigger level
Analog output	0V to 5V based on infrared radiation
LEDs indicating output and power	
PCB size	3.2cm x 1.4cm

#### 3.5.3 Current sensor ACS712

**Fig 21:**
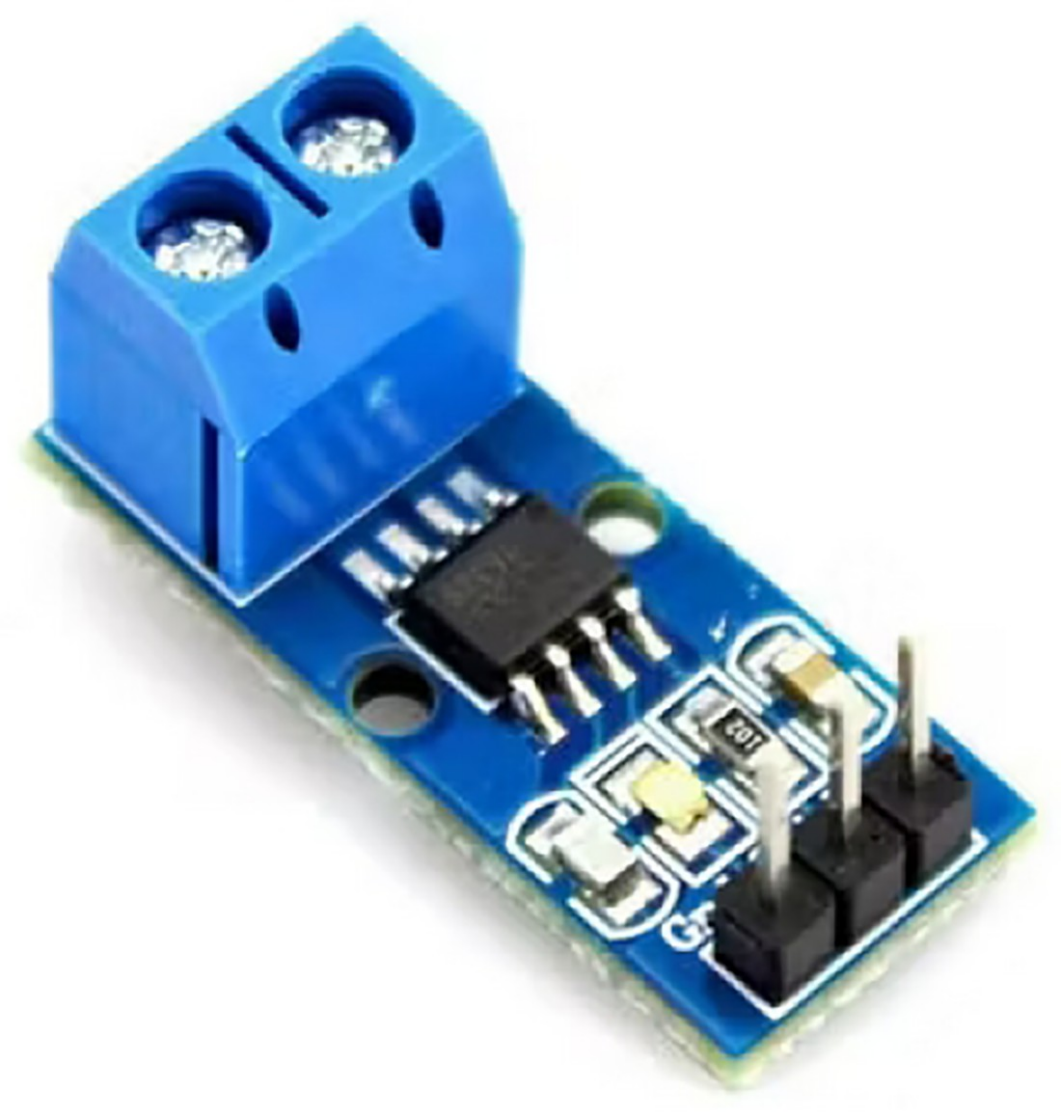
ACS712 module. Image credit to www.amazon.com.

The ACS712 module, see Fig 21, is a fully integrated, hall effect-based linear current sensor with 2.1kVRMS voltage isolation and an integrated low-resistance current conductor. Technical terms aside, the module is a current sensor that uses its conductor to calculate and measure the amount of current applied. ACS712 can detect both AC/DC currents. It can be used in a broader range of applications apart from electrical appliances. Table 3 lists the ACS712’s specifications.

**Table 3:** Technical specifications of the ACS712 sensor.

80kHz bandwith
66 to 185 mV/A output sensitivity
Low-noise analog signal path
Device bandwith is set via the new FILTER pin
1.2 mΩ internal conductor resistance
Total output error of 1.5% at TA = 25°C

#### 3.5.4 Digital humidity and temperature sensor SHT10

**Fig 22:**
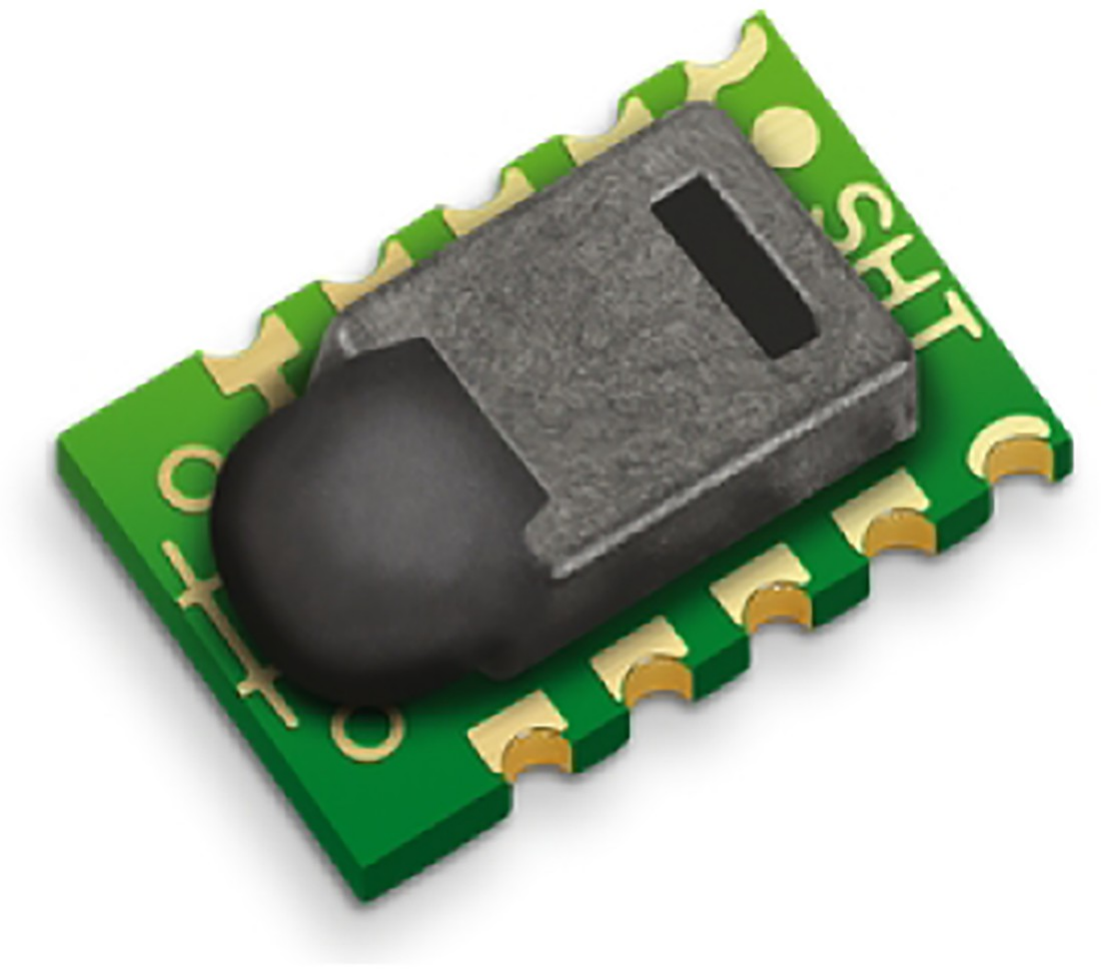
SHT10 module. Image credit to www.sensirion.com.

The SHT10 module, see Fig 22, is one of the SHT1x series of humidity sensors (including SHT10, SHT11, and SHT15) manufactured by Sensirion is compact, surface-mountable devices designed to measure relative humidity. These sensors combine sensor elements and signal processing in a small form factor, delivering a fully calibrated digital output. By utilizing a unique capacitive sensor element for humidity measurement and a band-gap sensor for temperature measurement, these sensors employ CMOSens© technology to ensure exceptional reliability and long-term stability. Moreover, the humidity sensors are seamlessly integrated with a 14-bit analog-to-digital converter and a serial interface circuit. It produces high-quality signals, rapid response times, and immunity to external disturbances such as electromagnetic interference (EMC). Each SHT1x humidity sensor undergoes individual calibration within a precise humidity chamber. The calibration coefficients are programmed into the chip’s OTP (one-time programmable) memory. These coefficients are employed for internal calibration of the sensor signals. With a two-wire serial interface and built-in voltage regulation, integrating the SHT1x sensors into systems is effortless and efficient. These sensors are supplied in a surface-mountable LCC (leadless chip carrier) package, suitable for standard reflow soldering processes and approved for use. Table 4 lists the SHT10’s specifications.

**Table 4:** Technical specifications of the SHT10 sensor.

Typ. relative humidity accuracy	4.5% RH
Operating relative humidity range	0 to 100% RH
Response humidity time	8s
Calibration certificate	Factory calibration
Typ. temperature accuracy	0.5°C
Operating temperature range	-40 to 125°C
Response temperature time	5s
Supply voltage	2.4V to 5.5V
Average supply current	28 uA
Interfaces	Sensibus
Dimension	7.5 x 4.9 x 2.6mm

#### 3.5.5 Arduino Uno R3

**Fig 23:**
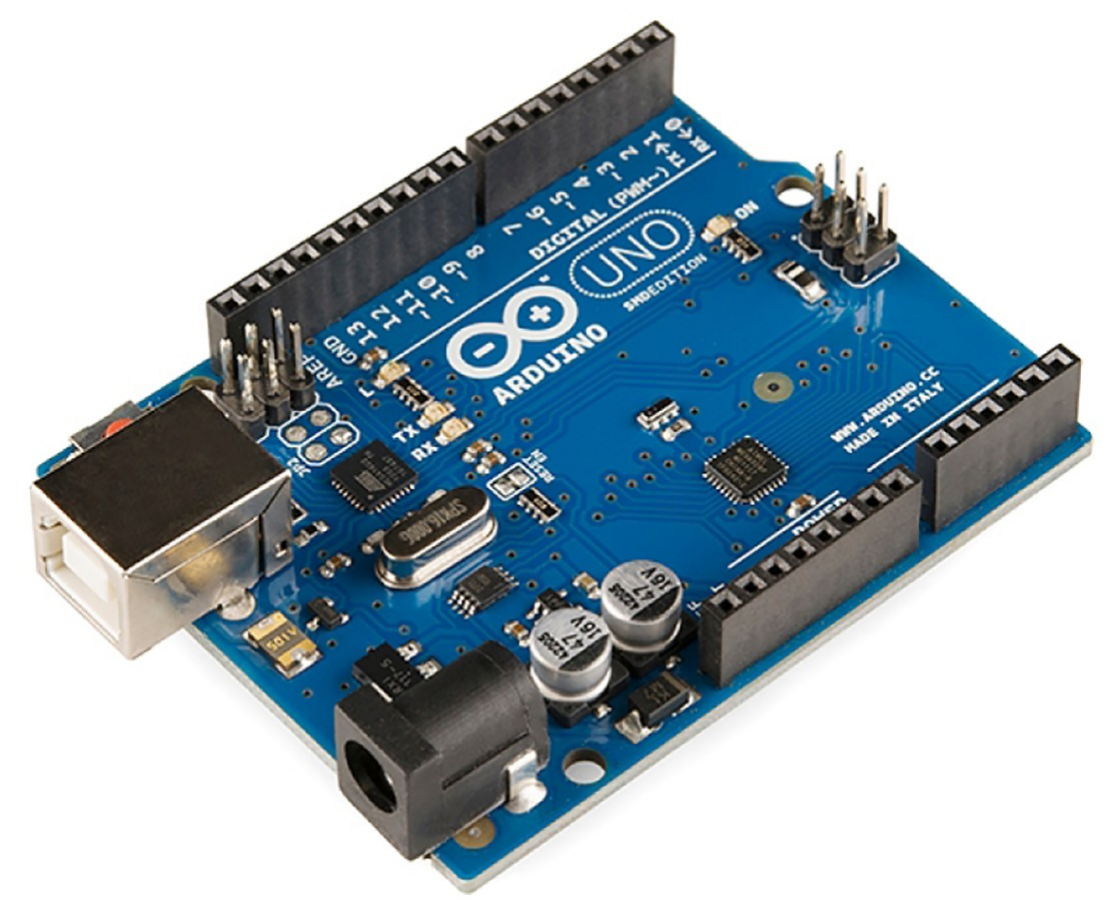
Uno R3 board. Image credit to www.wikipedia.com.

The Arduino Uno R3, see Fig 23, is a microcontroller board based on the ATmega328P. It provides all the necessary components to support the microcontroller, simply requiring a connection to a PC via a USB cable and a power source such as an AC-DC adapter or a battery to get started. “Uno” translates to “one” in Italian, signifying its significance as the board accompanying the release of Arduino’s IDE 1.0 software. The R3 version of the Arduino Uno is the board’s third and most recent iteration. The Arduino board and the IDE software are considered the standard versions of Arduino and continue to evolve with new releases. The Uno board serves as the primary USB-Arduino board in a series and represents the reference model for the Arduino platform. Table 5 lists the Uno R3’s specifications.

**Table 5:** Technical specifications of the Uno R3 board.

Microcontroller	ATmega328P
SKU	A000066
USB connector	USB-B
Built-in LED pins	13
Digital I/O pins	14
Analog input pins	6
PWM pins	6
UART, I2C, SPI communication	Yes
I/O Voltage	5 V
DC Current per I/O Pin	20 mA
Power Supply Connector	Barrel Plug
Main Processor	ATmega328P 16 MHz
USB-Serial Processor	ATmega16U2 16 MHz
Memory	2KB SRAM, 32KB FLASH, 1KB EEPROM
Dimension (Width x Length)	53.4mm x 68.6mm

#### 3.5.6 Arduino Mega 2560 Rev3

**Fig 24:**
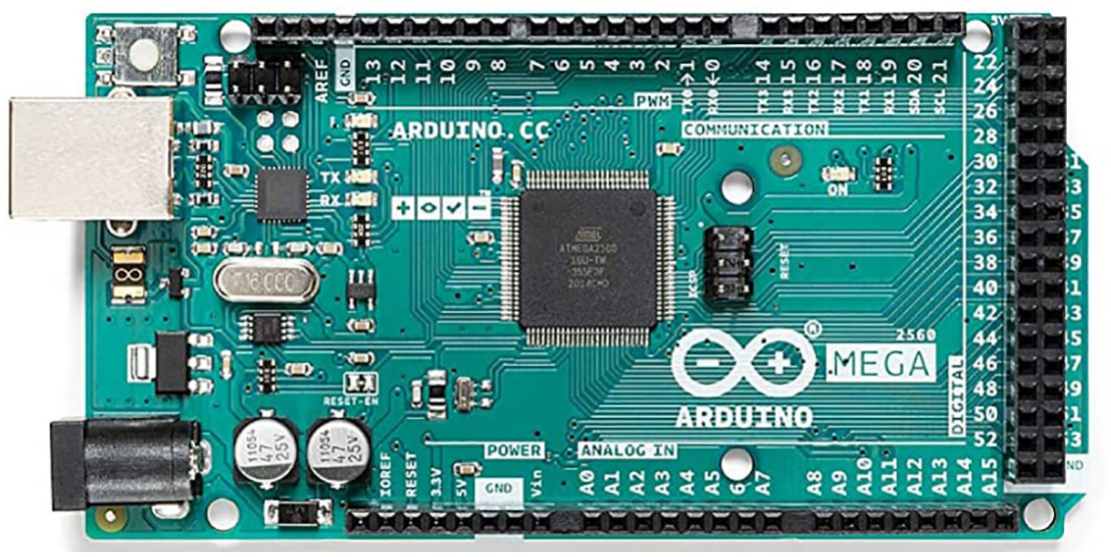
Mega 2560 Rev3 board. Image credit to www.amazon.com.

The Arduino Mega 2560 Rev3, see Fig 24, is a microcontroller board that utilizes the ATmega2560 as its core. It boasts an extensive array of features, including 54 digital input/output pins (with 15 capable of PWM output), 16 analog inputs, 4 UARTs for hardware serial communication, a 16 MHz crystal oscillator, a USB connection, a power jack, an ICSP header, and a reset button. This board provides everything required to support the microcontroller, enabling easy connectivity to a computer via USB or power supply through an AC-to-DC adapter or battery. The Arduino Mega 2560 is fully compatible with shields designed for the Uno, Duemilanove, or Diecimila boards, offering a versatile platform for various projects. The Arduino Mega 2560 Rev3 (R3) is an excellent choice when searching for a microcontroller board. It caters to various applications, particularly in small-scale do-it-yourself projects such as robotics and mini-computers. In the realm of 3D printing, it can serve as a remote 3D printer server (e.g., with OctoPrint) or function as a control board for a printer, providing ample possibilities for customization and control. Table 6 lists the Mega 2560 Rev3’s specifications.

**Table 6:** Technical specifications of the Mega 2560 Rev3 board.

Microcontroller	ATmega2560
SKU	A000067
USB connector	USB-B
Built-in LED pins	13
Digital I/O pins	54
Analog input pins	16
PWM pins	15
UART, I2C, SPI communication	Yes
I/O Voltage	5 V
Supported battery	9 V battery
DC Current per I/O Pin	20 mA
Power Supply Connector	Barrel Plug
Main Processor	ATmega2560 16 MHz
USB-Serial Processor	ATmega16U2 16 MHz
Memory	8KB SRAM, 256KB FLASH, 4KB EEPROM
Dimension (Width x Length)	53.3mm x 101.5mm

#### 3.5.7 Arduino ESP8266 Wifi Shield

**Fig 25:**
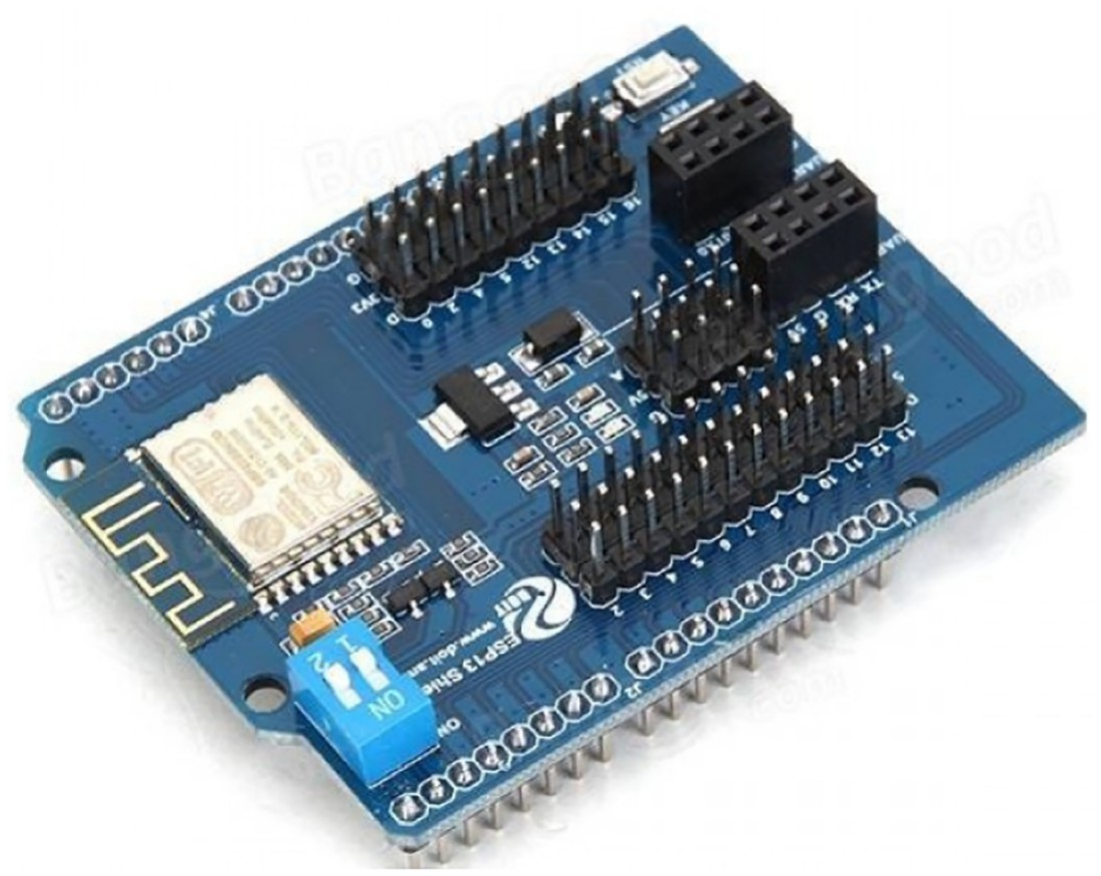
ESP8266 WIFI Shield. Image credit to www.robotpark.com.

ESP8266 WIFI Shield, see Fig 25, is a cost-effective and highly integrated UART-WiFi module for IoT applications. It has excellent dimensions and ULP technology compared to other similar modules. The module is designed for mobile devices and the Internet of Things (IoT). This WiFi Shield is based on ESP-12F, which is the new version of the ESP-12 with the WiFi chip ESP8266. With this Shield, you can make your Arduino easy to connect to the network and control your device anywhere. Arduino ESP-13 WiFi Shield ESP8266 Compatible With Arduino Uno, Mega 2560. It can also be used as an independent development board, download the official AT commands firmware and NodeMCU open source firmware. Table 7 lists the ESP8266’s specifications.

**Table 7:** Technical specifications of the Arduino ESP8266 Wifi Shield.

Wireless 802.11 b / g / n standards
WiFi current: continuous transmission: 70mA (200mA MAX), Standby: <200uA
Wireless transmission rate: 110-460800bps
Support for STA / AP two modes of operation
Built-in TCP / IP protocol stack
Supports standard TCP / UDP Server and Client
Serial port baud rate support: 1200/2400/4800/9600/19200/38400/57600/74800/115200 bps
Serial data bits: 5 / 6 / 7 / 8 bits
Serial port stop bits: 1/2 bit
Leads out Arduino Pin 2 / 3 / 4 / 5 / 6 / 7 / 8 / 9 / 10 / 11 / 12 / 13
Leads out ESP8266 GPIO 0/2/4/5/9/10/12/13/14/15/16/ADC/EN/UART TX/UART RX
KEY multiplex configuration function keys
RESET function
Two DIP switches enable switching Arduino and ESP8266 serial port expansion
Working temperature: -40 ℃ ∼ + 125 ℃

**Fig 26:**
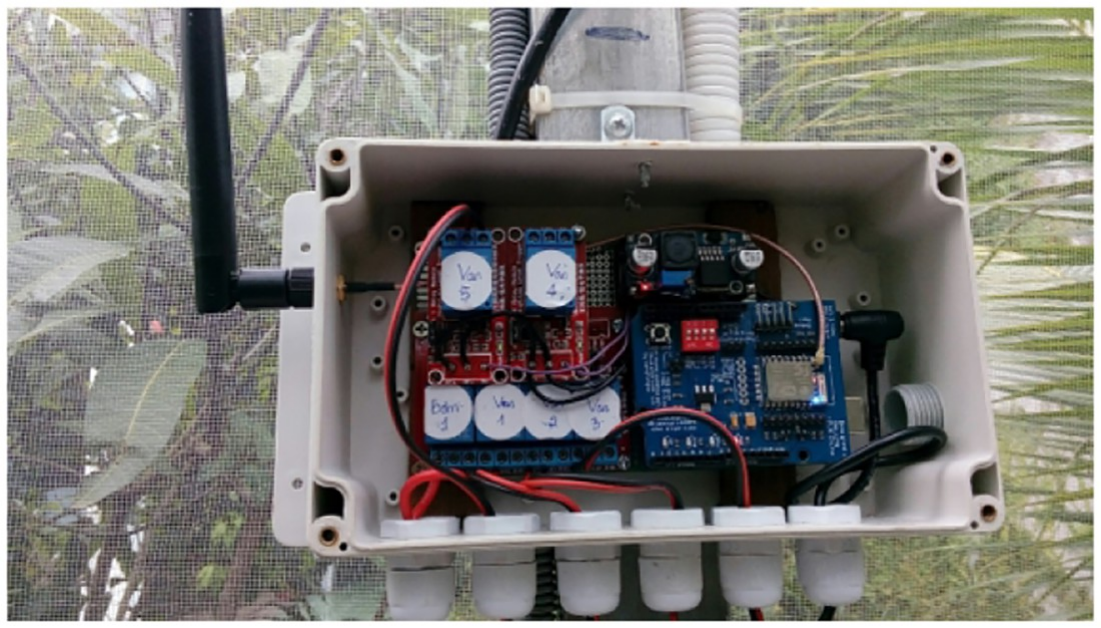
Our self-designed IoT box.

The IoT box, as illustrated in Fig 26, serves as the central hub or gateway for orchestrating and connecting numerous IoT devices within a network. Its primary function is to enable monitoring, control, and data exchange between these devices and the cloud platform or user interface. Through the consolidation of control boards and the provision of various connectivity options, the IoT box simplifies the process of integrating and managing IoT devices. It is worth noting that we acquired a suitable IoT box from a local store for our implementation. Depending on local availability and specific requirements, additional IoT boxes can be procured for interesting implementations.

### 3.6 Graphical user interface

The Graphical User Interface (GUI) for our smart greenhouse encompasses a local web interface and an Android mobile application, providing users with convenient and intuitive ways to interact with the system.

The local web interface serves as a user interface accessible through a web browser on a local network. It allows users to monitor and control the various aspects of the smart greenhouse from their desktop or laptop computers. Users can access real-time data through the web interface, such as temperature, humidity, soil moisture levels, and system status. They can also configure and adjust settings related to irrigation schedules, lighting, ventilation, and other parameters. The web interface offers a responsive and user-friendly design, ensuring a seamless experience for users accessing it from different devices.

The Android mobile application provides an additional means of interacting with the smart greenhouse, offering mobility and convenience. Users can download and install the application on their Android smartphones or tablets. The mobile application provides similar functionality to the web interface, allowing users to monitor sensor data, control devices, and adjust settings remotely. With the mobile application, users can access and manage the smart greenhouse while on the go, providing real-time updates and control at their fingertips.

Both the local web interface and the Android mobile application are designed to emphasize user experience, ensuring ease of use, visual appeal, and intuitive navigation. These interfaces provide seamless integration with the smart greenhouse system, enabling users to monitor, manage, and optimize the greenhouse environment and operations.

#### 3.6.1 Local web interface

**Fig 27:**
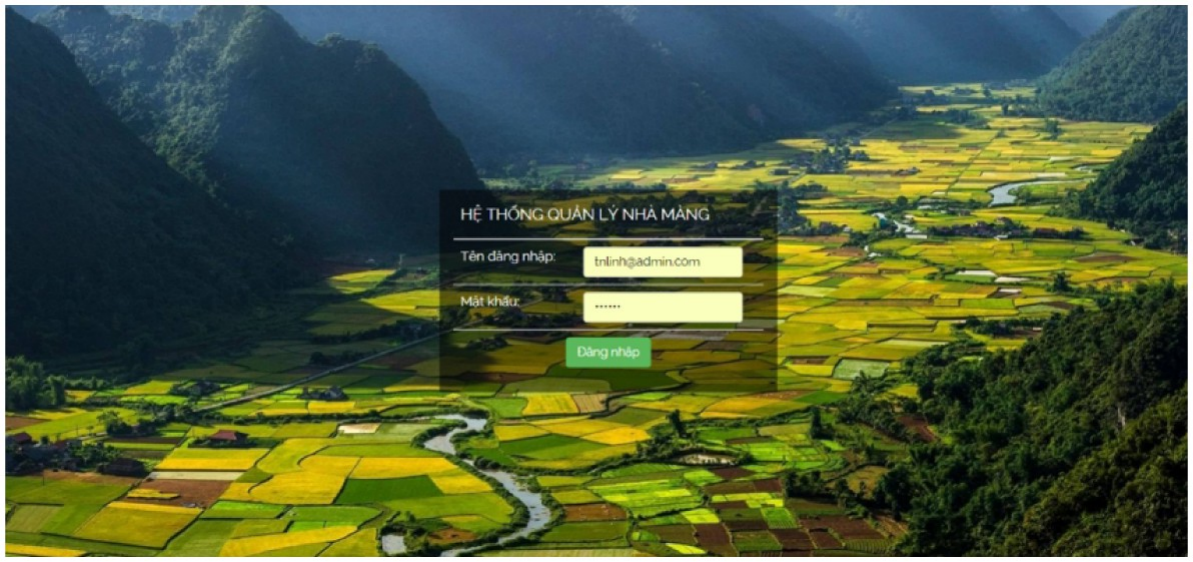
Smart greenhouse login page.

**Fig 28:**
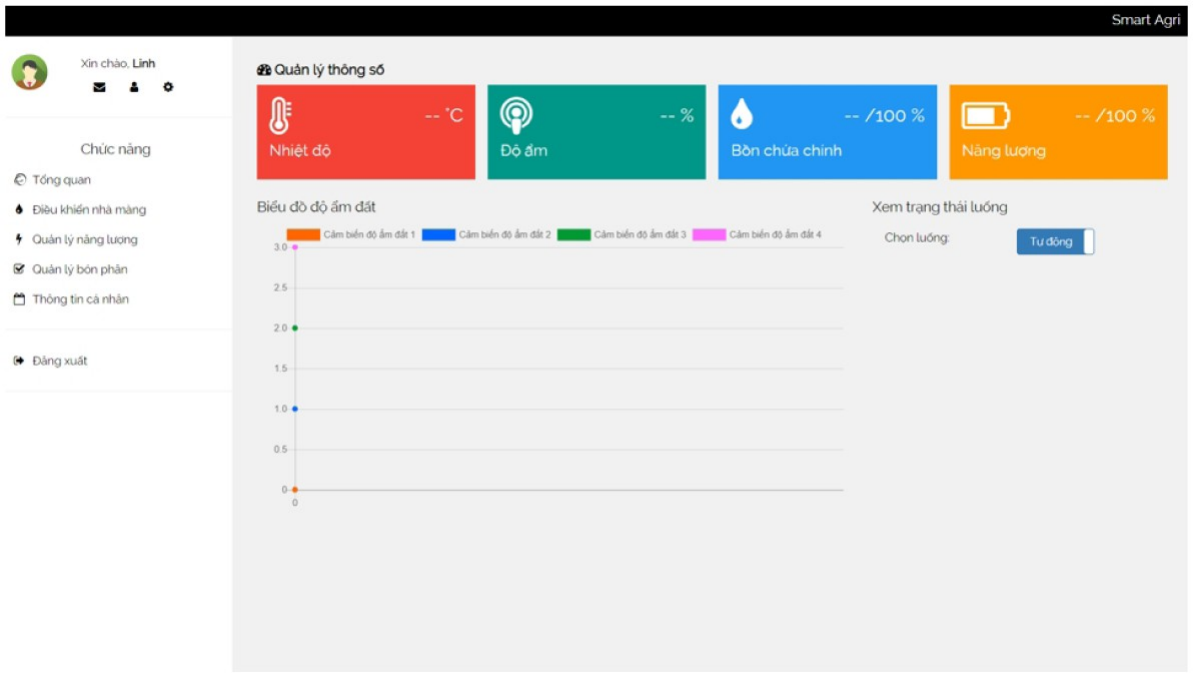
Smart greenhouse home page.

**Fig 29:**
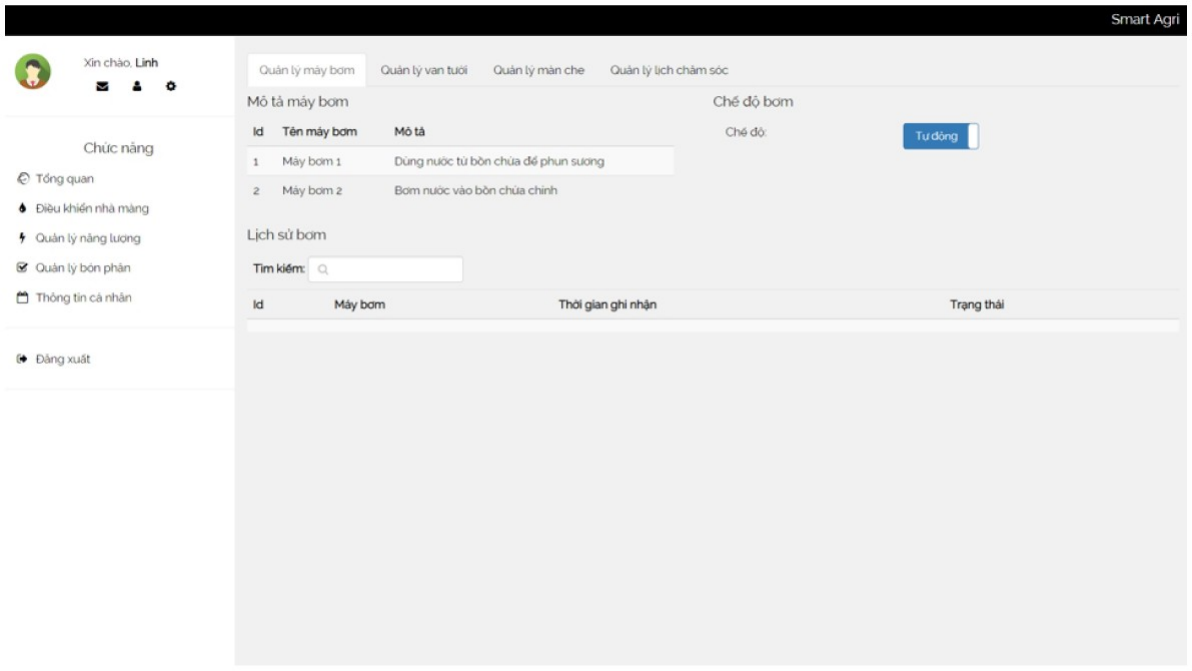
Water pump management page.

**Fig 30:**
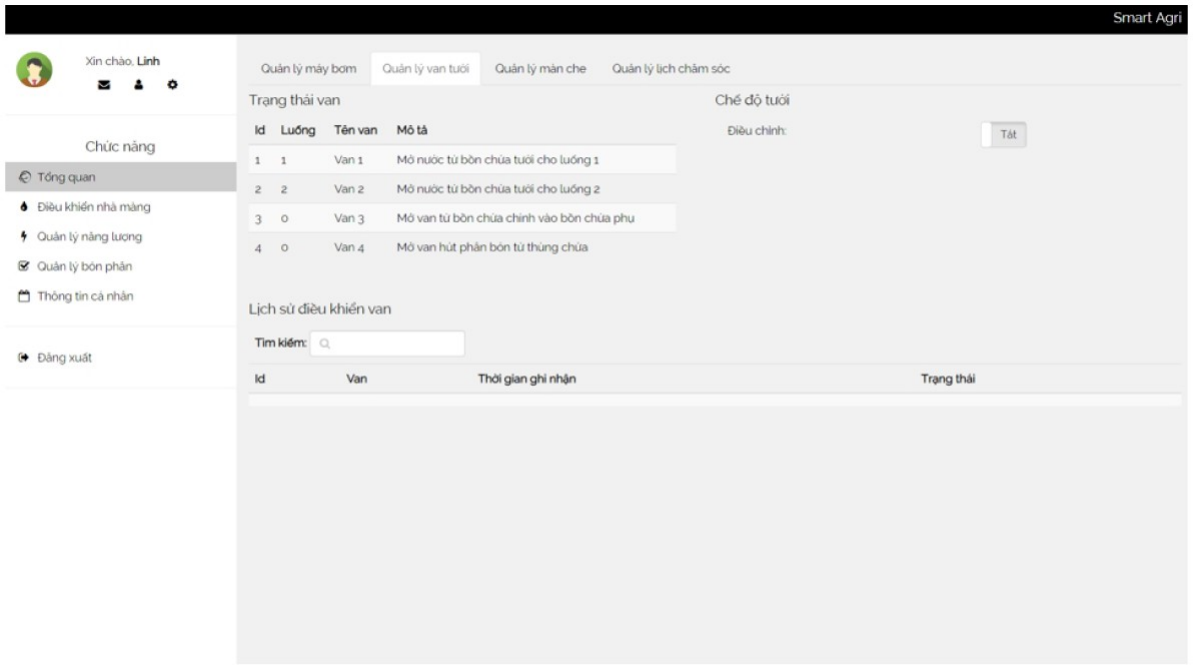
Irrigation valve management page.

**Fig 31:**
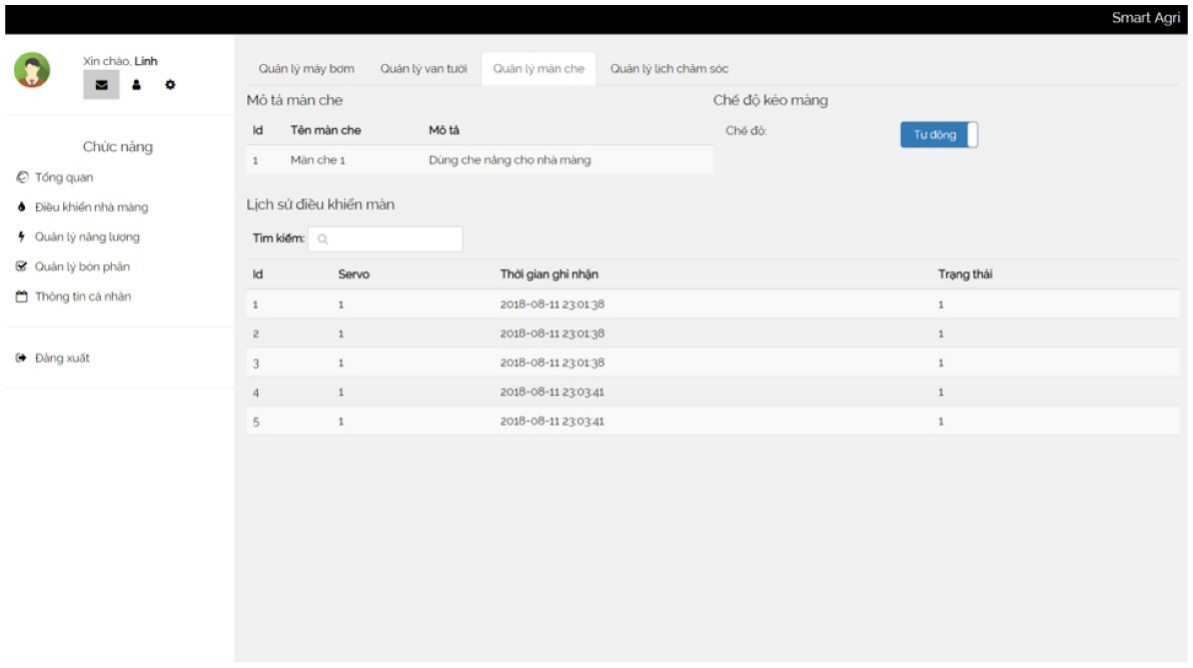
House’s dome management page.

**Fig 32:**
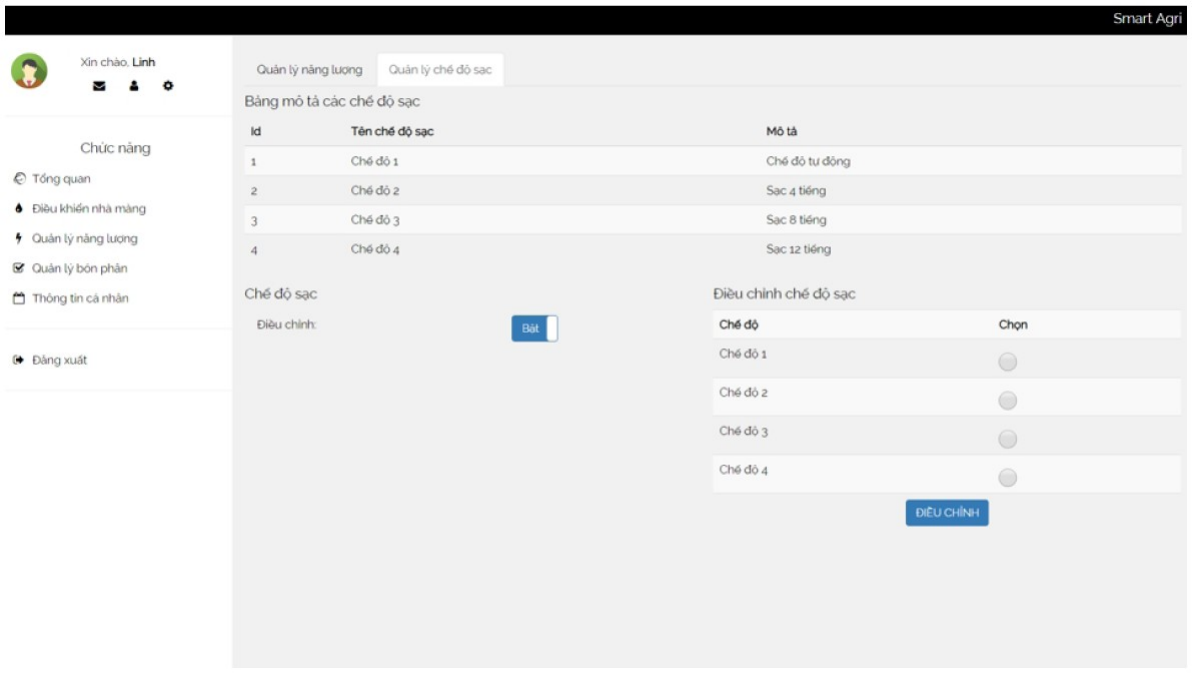
Power and charging management page.

**Fig 33:**
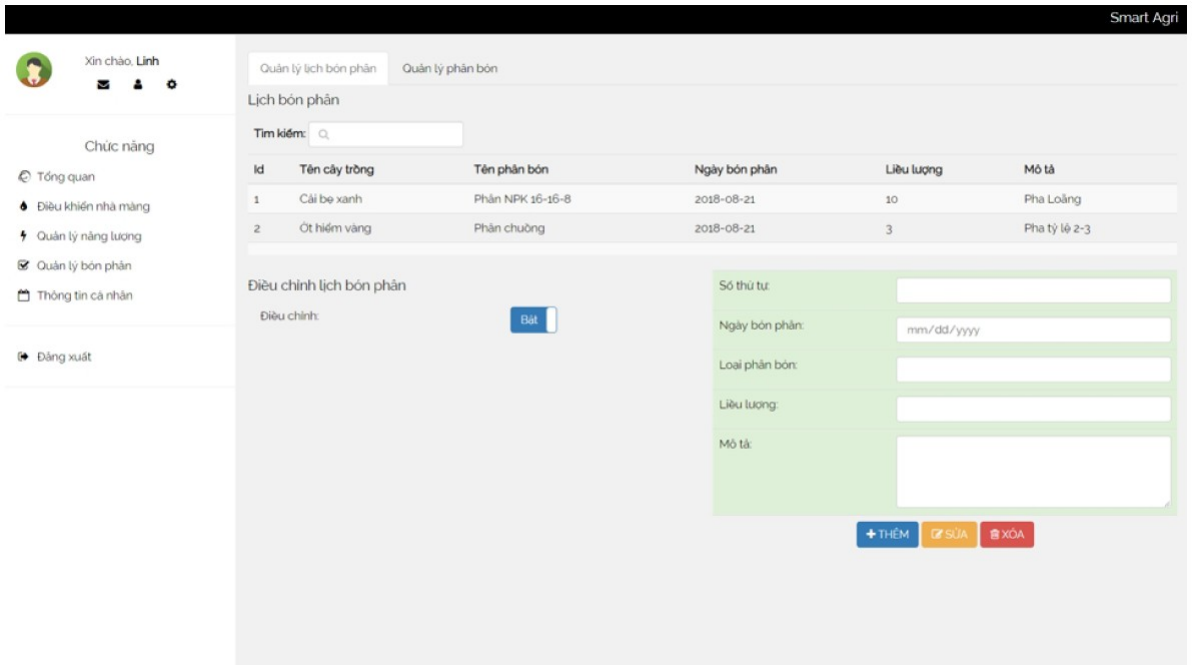
Fertilizer schedule management page.

Upon accessing the local web interface, users have an intuitive dashboard that overviews the greenhouse’s key parameters and status. They can easily view real-time data such as temperature, humidity, water pump, greenhouse roof, and soil moisture, enabling them to monitor the current conditions inside the greenhouse at a glance. In addition to real-time data, the web interface offers comprehensive control features. Users can configure and adjust irrigation schedules, control house roofs, manage ventilation, and monitor the status of devices such as pumps and valves. This level of control allows users to fine-tune the greenhouse environment according to the specific requirements of different plants and optimize their growth conditions. The web interface also provides graphical representations of historical data and trends, allowing users to analyze and track the greenhouse’s performance over time. This data visualization helps users identify patterns, make informed decisions, and fine-tune their cultivation strategies for maximum efficiency and productivity. We present several screenshots of our local web interface in Figs 27–33.

Fig 27 depicts the login page, where users are required to enter their correct username and password to access the system. This essential security measure ensures that only authorized individuals can interact with the smart greenhouse’s control interface.

Upon successful login, Fig 28 presents the main control panel. This central hub empowers users to configure and monitor a comprehensive array of vital parameters, including temperature, air humidity, water level, fertilizer level, and energy consumption. It serves as the nerve center for overseeing and fine-tuning the environmental conditions critical for optimal crop growth.

Fig 29 is dedicated to the management of all water pumps employed in the system. Users have the flexibility to configure watering schedules, choosing between automatic and manual modes. This level of control ensures that water distribution aligns precisely with the specific needs of different crops.

Similarly, Fig 30 focuses on the management of irrigation valves. Users can configure these valves individually or in groups, specifying whether they should operate in automatic or manual mode. This fine-grained control ensures precise and efficient irrigation tailored to the requirements of various plant types.

Fig 31 provides a dedicated interface for configuring the greenhouse’s dome. Users can specify conditions under which the dome should open or close, optimizing ventilation and climate control to suit changing weather conditions and crop preferences.

In Fig 32, users can monitor the status of all batteries and track energy consumption throughout the system. This real-time insight enables efficient energy management and ensures uninterrupted operation of the smart greenhouse.

Fig 33 is the control center for configuring fertilizer schedules. Users can define when and how specific fertilizers should be mixed into the water source, streamlining the process of nutrient delivery to the plants.

These intuitive and comprehensive web-based interfaces provide users with the tools they need to efficiently manage and optimize the smart greenhouse’s operations, promoting precision agriculture and contributing to enhanced crop productivity and resource efficiency.

#### 3.6.2 Android mobile application

**Fig 34:**
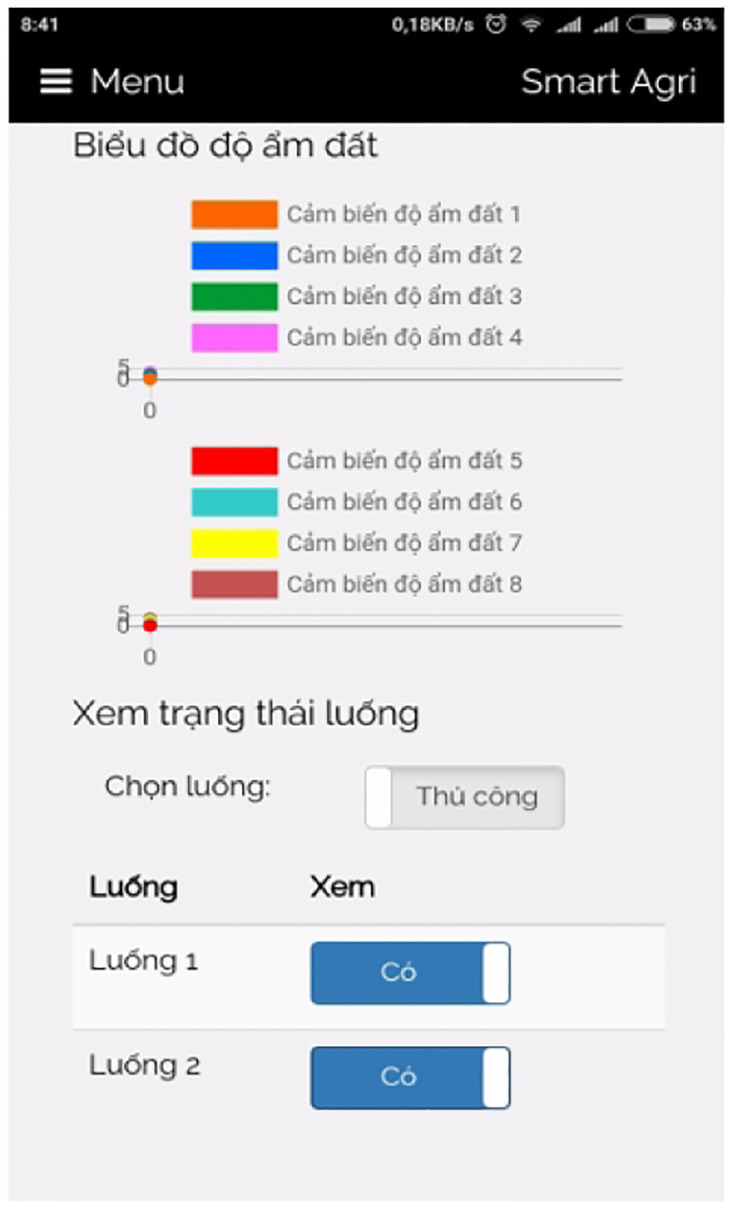
Moisture management page.

**Fig 35:**
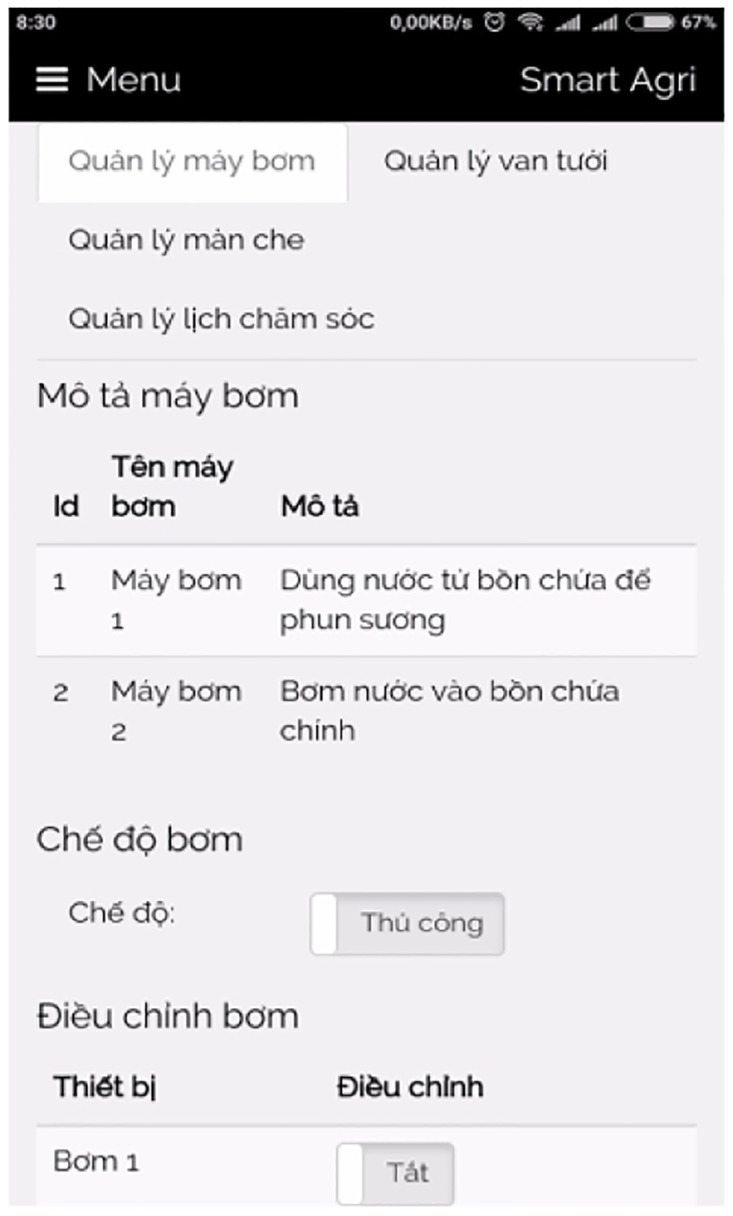
Water pump management page.

**Fig 36:**
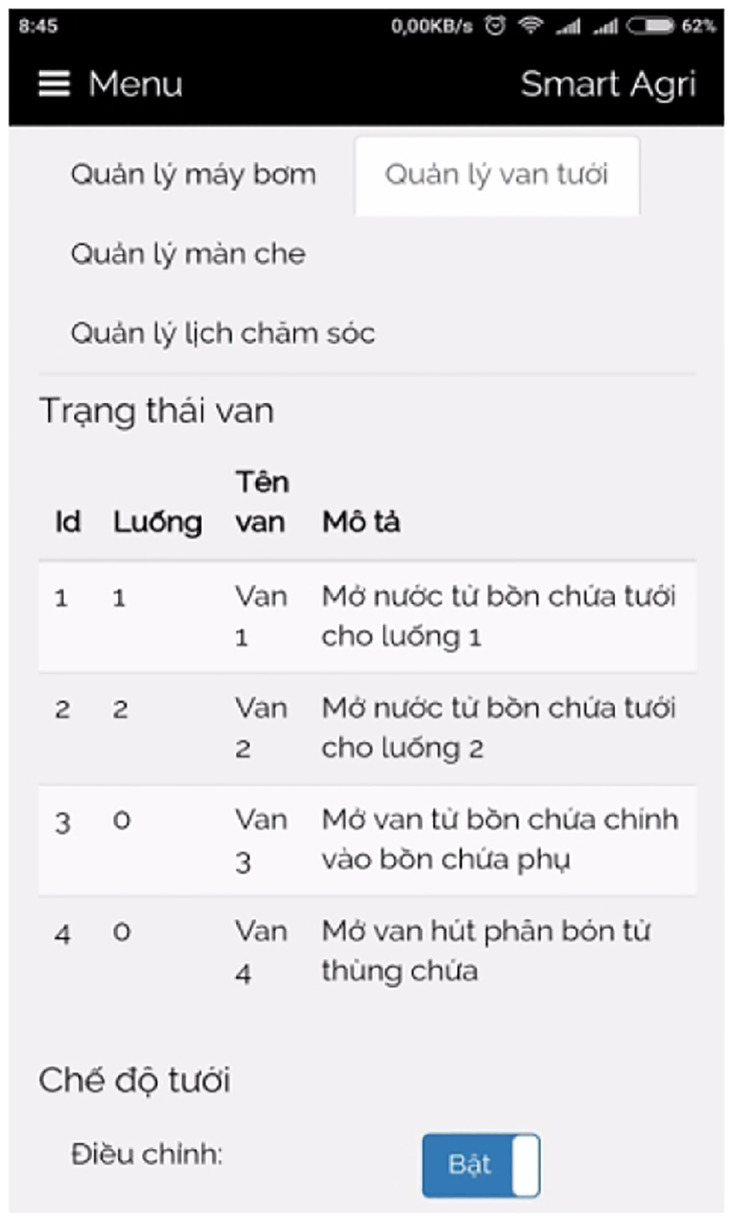
Irrigation valve page.

**Fig 37:**
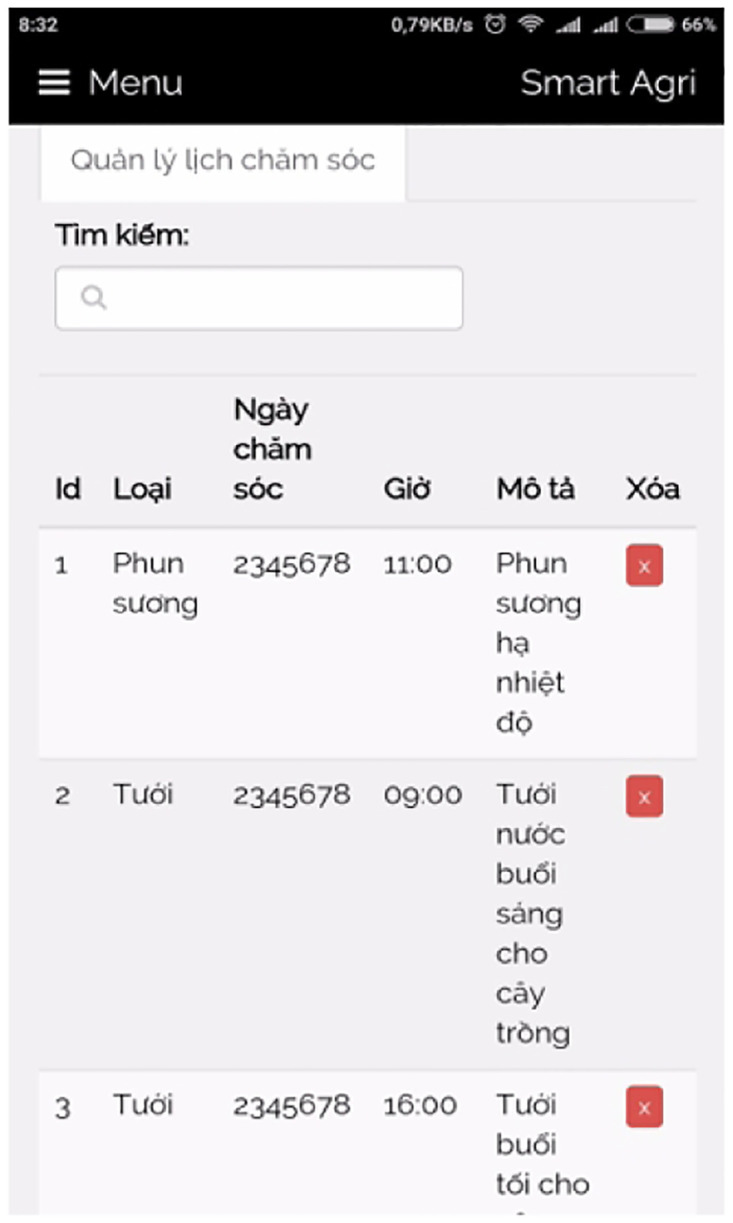
Irrigation schedule management page.

**Fig 38:**
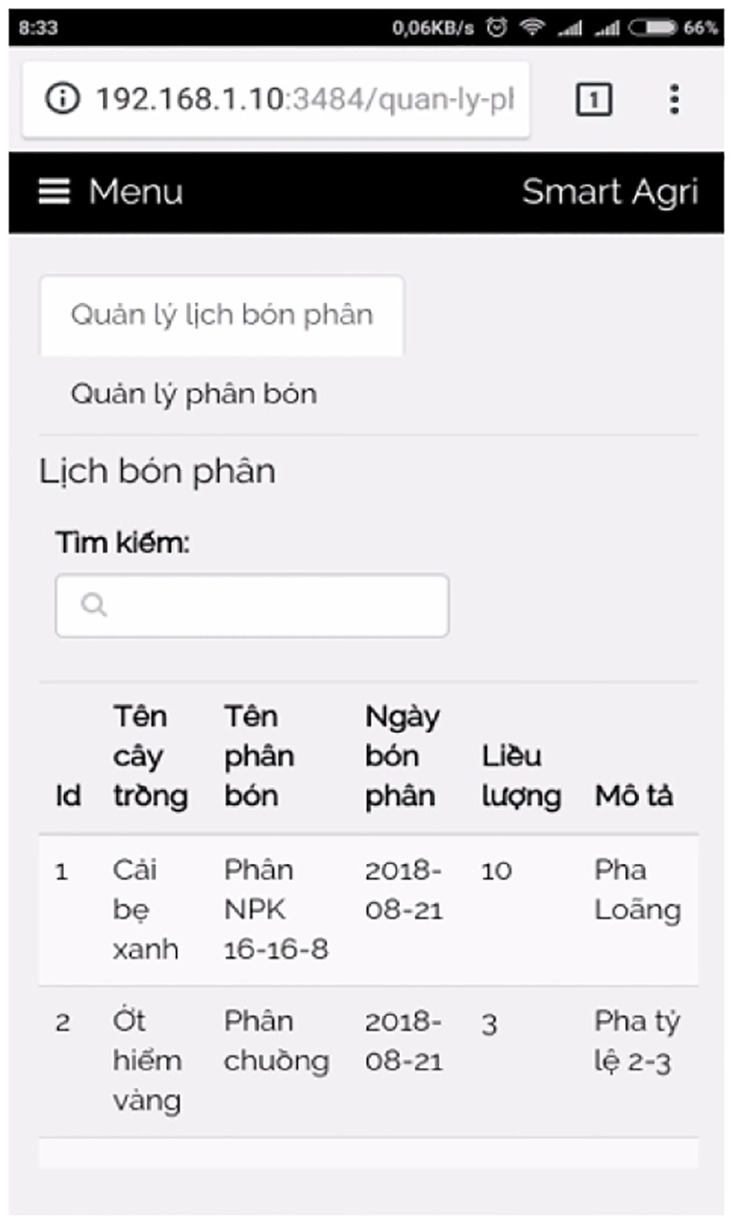
Fertilizer schedule management page.

The mobile application designed for our innovative greenhouse offers a convenient and portable solution for users to access and manage the system from their Android smartphones or tablets. It provides a seamless and user-friendly interface allowing users to monitor and control their greenhouse operations while moving. Upon launching the mobile application, users are greeted with a visually appealing and intuitive interface. The mobile application allows users to remotely control and adjust various aspects of the greenhouse environment. Users can easily configure irrigation schedules, set desired temperature and humidity levels, adjust dome settings, and control ventilation systems. The app provides a user-friendly interface with intuitive controls and sliders, enabling users to quickly and precisely adjust greenhouse conditions with just a few taps. Real-time data updates are displayed prominently within the mobile application, allowing users to stay informed about the current state of the greenhouse. They can monitor sensor readings, view charts and graphs illustrating historical data trends, and receive notifications and alerts for important events such as abnormal temperature fluctuations or low water levels. This real-time data access empowers users to make timely decisions and take appropriate actions to ensure optimal plant growth conditions. We present several screenshots of our Android mobile interface in Figs 34–38.

The functionalities showcased in those figures closely mirror those discussed in the web interface detailed in the preceding section. The primary distinction lies in the provision of an Android mobile interface, offering users the flexibility to access and control the smart greenhouse system on-the-go. It is advisable for readers to consult the illustrations provided in Section 3.6.1 for an in-depth understanding of these functions, as they remain consistent across both the web and mobile interfaces. This coherence ensures that users can seamlessly transition between the two interfaces while enjoying a unified and accessible experience, whether they choose to engage with the system via a web browser or the Android mobile application.

## 4 Remarks and discussion

Combining self-constructed smart greenhouse infrastructure, IoT technology integration, a web interface, and an Android mobile application represents a remarkable advancement in cultivating Brassica Juncea—a versatile and nutritious crop. This sophisticated system offers multifaceted benefits by enabling precise monitoring, control, and management of the greenhouse environment, ultimately leading to optimal growth conditions and heightened productivity for Brassica Juncea. The successful accomplishment of our research objectives in this study has yielded transformative impacts within smart greenhouse technology and IoT research domains. By effectively attaining these objectives, we have propelled critical aspects of smart greenhouse technology and IoT integration to new heights, resulting in profound advancements in the field.

Our objectives, including implementing automated fertilization, watering, and meticulous fertilizer record-keeping, represent pivotal contributions to the evolution of precision agriculture within intelligent greenhouse systems. These automated processes have significantly enhanced operational efficiency, ensuring the accurate and precise application of fertilizers and water resources. It, in turn, translates into augmented crop yields and more effective resource management. Furthermore, integrating weather parameter management and providing data-driven recommendations for weather, watering, and fertilization schedules have exemplified the fusion of real-time environmental data analysis and sophisticated decision-making algorithms. This integration enhances the intelligence and adaptability of intelligent greenhouse systems, empowering them to respond dynamically and optimize ecological conditions based on current weather patterns and the specific requirements of the crops being cultivated. In essence, our research represents a significant leap forward in the intelligent cultivation of Brassica Juncea. It underscores the broader potential of smart greenhouse technology and IoT integration in revolutionizing modern agriculture. These innovations promise to enhance crop productivity and contribute to sustainable and resource-efficient farming practices, thereby addressing critical challenges in food production and environmental sustainability.

The focus on energy management has played a pivotal role in promoting sustainable and efficient operation of smart greenhouse systems. This research has advanced the utilization of clean and renewable energy in agriculture by monitoring and optimizing energy usage and integrating renewable energy sources such as solar cells. It has paved the way for further exploration of energy-efficient solutions and renewable energy integration in IoT-based agricultural systems. The fertilizer management objective, encompassing scheduling, categorization, and record-keeping of fertilizer applications, has contributed to developing improved methods for managing and tracking fertilizer usage. It has optimized nutrient delivery to plants while minimizing environmental impacts.

Moreover, the objective of personal information management has underscored the significance of secure user data management and access control in IoT-based agricultural applications. It has laid the foundation for robust security measures, safeguarding sensitive information and ensuring user trust in IoT-enabled smart greenhouse systems. In conclusion, accomplishing the research objectives outlined in this study has provided valuable insights and propelled the advancement of smart greenhouses and IoT research. It has expanded our understanding and capabilities in utilizing IoT technologies for precision agriculture and environmental control within greenhouse settings.

## 5 Conclusion

This research marks a significant milestone in smart greenhouse technology and IoT integration. The research team has successfully achieved the initial objectives, which encompassed the development of a multifunctional system, including automatic fertilization, precise watering, and comprehensive fertilizer record-keeping. Beyond these core functions, the system provides invaluable weather, watering, and fertilization recommendations, adding intelligent decision-making support. Our endeavors have yielded a user-friendly interface accessible through web and Android mobile devices, enabling seamless interaction with the smart greenhouse system. Key features include real-time weather parameter monitoring, automated fogging decisions, and soil moisture parameter tracking with automatic irrigation management. The system also empowers users to monitor energy usage and charging progress while recording their charging mode preferences. Additionally, the system boasts a fertilizer management module that facilitates scheduling, categorizing, and meticulous record-keeping of fertilizer applications. Our research has led to the successful development of this comprehensive smart greenhouse system, complete with automation, data recording, decision-making support, and user management capabilities. It is crucial to underscore that our work represents a pioneering effort, being the first to propose and implement a complete smart greenhouse IoT solution dedicated to the development of Brassica Juncea, solidifying our unique contribution to the field. These advancements hold immense promise for revolutionizing crop cultivation and play a pivotal role in addressing the pressing challenges of food production, resource conservation, and environmental sustainability. As such, our research sets the stage for continued innovation in smart agriculture and greenhouse technology.
